# Effects of the spatial resolution of the Virtual Epileptic Patient on the identification of epileptogenic networks

**DOI:** 10.1162/imag_a_00153

**Published:** 2024-05-08

**Authors:** Jean-Didier Lemaréchal, Paul Triebkorn, Anirudh Nihalani Vattikonda, Meysam Hashemi, Marmaduke Woodman, Maxime Guye, Fabrice Bartolomei, Huifang E. Wang, Viktor Jirsa

**Affiliations:** Aix-Marseille Université, Institut National de la Santé et de la Recherche Médicale, Institut de Neurosciences des Systèmes (INS) UMR1106, Marseille, France; Aix-Marseille Université, CNRS, CRMBM, Marseille, France; APHM, Timone University Hospital, CEMEREM, Marseille, France; APHM, Epileptology and Clinical Neurophysiology Department, Timone Hospital, Marseille, France

**Keywords:** Virtual Epileptic Patient (VEP), epileptogenic network, stereotactic electroencephalography, neural field model, neural mass model, inference

## Abstract

Digital twins play an increasing role in clinical decision making. This study evaluates a digital brain twin approach in presurgical evaluation, the Virtual Epileptic Patient (VEP), which estimates the epileptogenic zone in patients with drug-resistant epilepsy. We built the personalized digital brain twins of 14 patients and a series of synthetic dataset by considering different spatial configurations of the epileptogenic and/or propagation zone networks (EZN and PZN, respectively). Brain source signals were simulated with a high spatial resolution neural field model (NFM) composed of 81942 nodes, embedding both long-range (between brain regions) and short-range (within brain regions) coupling. Brain signals were then projected to stereotactic electroencephalographic (SEEG) contacts with an accurate forward solution. An inversion procedure based on a low spatial resolution neural mass model (NMM) composed of 162 nodes was applied to estimate the excitability of each region in each simulation. The ensuing estimated EZN/PZN was compared to the simulated ground truth by means of classification metrics. Overall, we observed correct but degraded performance when using an NMM to estimate the EZN from data simulated with an NFM, which was significant for the simplest spatial configurations. We quantified the reduced performance and demonstrated that the oversimplification of the forward problem is its principal cause. We showed that the absence of local coupling in the NMM affects the inversion process by an overestimation of the excitability, representing a significant clinical impact when using this procedure in the context of presurgical planning. In conclusion, this study highlighted the importance to shift from an NMM towards a full NFM modeling approach for the estimation of EZN, with a particularly relevant need when considering the most complex clinical cases.

## 1 Introduction

### 1.1 Introduction of the VEP

Epilepsy is a common neurological disorder characterized by recurrent seizures (Fisher et al., 2014;Noebels et al., 2012). Epileptic seizures very often involve networks of connected brain regions (Bartolomei et al., 2017;Besson et al., 2017) and present complex spatiotemporal brain dynamics (da Silva et al., 2003;Kalitzin et al., 2019;Saggio et al., 2020). For patients with pharmaco-resistant focal epilepsy, resective surgery is one invasive solution to become seizure free. The goal of this strategy is to remove a sufficient and minimal set of epileptognic regions which are responsible for seizure initiation (Jehi, 2018;Lüders et al., 2006;Rosenow & Lüders, 2001). Stereotactic electroencephalographic (SEEG) is one of the tools used during presurgical planning in order to identify those early recruited regions (Bartolomei et al., 2018;Bernabei et al., 2023;Cardinale et al., 2019;Mercier et al., 2022). This procedure consists in implanting a series of electrodes in the brain of one patient. The location of these electrodes is chosen to test and validate a clinical hypothesis derived from other diagnostic techniques (imaging, EEG, clinical tests). Brain activity is recorded continuously at the milliseconds time scale along the contacts of the depth electrodes separated from a few millimeters. These very high spatiotemporal resolved signals may record different kinds of typical epileptic activity (interictal epileptiform discharges, spontaneous or induced seizures), which are then used to infer the location of the targeted regions for the surgery (Lagarde et al., 2019). To better estimate the localization of the seizure onset zone based on SEEG recordings, many data-driven approaches based on the analysis of functional connectivity have been developed to identify and characterize the dynamics of interictal and ictal brain activity, as well as transitions between them (Wendling et al., 2010), such as the epileptogenicity index (Bartolomei et al., 2008). To complement and extend this approach, a series of computational models have been developed in order to capture specific dynamic features (Wendling et al., 2016). These models, either biophysical or more phenomenological, usually reduce these complex and non-linear dynamics to a set of coupled differential equations which are solved numerically to form solutions. Importantly, they represent an efficient tool to integrate multimodal data (structural, functional, pathophysiological) of one patient to obtain a personalized model (V. Jirsa et al., 2023). Moreover, when considering empirical data of one specific patient, the parameters of these models can be estimated based on inference techniques, such that the predictions of the model best fit the observations of the patient.

In this context, the Virtual Epileptic Patient (VEP) has been introduced as a personalized generative whole brain model of epilepsy spread (V. K. Jirsa et al., 2017;Wang et al., 2023). The VEP integrates the individual structural data of the patient (brain geometry, structural connectivity, location of SEEG electrodes) with a phenomenological model of epileptic activity (V. K. Jirsa et al., 2014). In particular, it implements a straightforward and efficient way of parameterizing the virtual epileptogenic network (Bartolomei et al., 2017) of the patient in terms of an epileptogenic zone network (EZN), a propagation zone network (PZN), and non-involved zones (NIZ). The distinction between these three networks follows the actual concept of a hierarchical organization taking place during a focal seizure within the brain of a patient with epilepsy. The EZN gathers brain regions with high epileptogenicity which are able to trigger seizures. After the initiation of a seizure, abnormal ictal activity may propagate to regions with lower epileptogenicity, the PZN. Finally, brain regions which do not show any significant change of their activity during a seizure are considered as NIZ. One major application of the VEP is the estimation of the EZN based on empirical SEEG recordings of spontaneous or stimulation-induced seizures (model inversion). Once the EZN has been accurately identified, the VEP can also reliably predict the spatiotemporal pattern of propagation (Proix et al., 2017;Wang et al., 2023). Notably, this specific VEP approach falls within a broader context of computational modeling methods (Bernabei et al., 2023;Lytton, 2008) that have been developed to estimate EZN and predict effect of therapy (Goodfellow et al., 2016;Kini et al., 2019;Olmi et al., 2019;Sinha et al., 2017). Given that currently, surgery does not systematically cure the patients (Cardinale et al., 2019), these studies show how modeling can bring significant contribution in planning therapeutic strategies tailored to the specificity of each patient and that collective efforts are still needed for a better clinical translation of these computational techniques (Litt, 2022).

### 1.2 Distinction between NMM and NFM

In this study, we focus the attention on two essential attributes of a VEP: the spatial model and the neuronal model.

The spatial model is either a Neural Mass Model (NMM) or a Neural Field Model (NFM) (Deco et al., 2008). In brief, the key difference between NMM and NFM is their spatial resolution. On the one hand, NMM is a brain model specified as a finite number of regions corresponding to macroscopic parts of the brain (a few cm^2^or cm^3^) and usually defined by a specific parcellation. In this study, we considered the VEP atlas (Wang et al., 2021) comprising 162 regions (73 cortical and 8 subcortical regions per hemisphere). These regions are represented as point sources interacting with each other through long-range connections (order of cm). On the other hand, NFM overcomes the spatial limitation of NMM by considering the brain as a spatially continuous network (V. K. Jirsa, 2009). This has at least two significant implications for NFM. First, in addition to a long-range (cm) coupling between distant macroscopic brain regions, that is, global connectivity, a short-range (mm) coupling, that is, local connectivity, can be modeled. This property is crucial for modeling seizure spread along cortical or subcortical surfaces (Liou et al., 2020;Martinet et al., 2017;Schlafly et al., 2022). And second, the projection of the simulated brain activity to the SEEG signals recorded at the electrode level, that is, the forward solution, can be modeled with better accuracy. Indeed, it allows to model brain sources as electrical dipoles and to consider their relative position and orientation with respect to the SEEG contacts, which is essential when computing electromagnetic propagation of electrical activity. In practical implementations, NFM is typically discretized on brain surfaces rather than being discretized point-wise, as is done in the NMM approach. In this study, we use 81924 vertices for the cortical surface. Necessarily, such very high spatial resolution of NFM, compared to the 162 nodes of NMM, comes with a significant additional computational cost.

In the present context, the neuronal model used at each node of the network is either the full Epileptor with 5 state variables (5D) in its original implementation (V. K. Jirsa et al., 2014) or its two-dimensional (2D) reduction (Proix et al., 2014).

### 1.3 Development of the VEP

Since the introduction of the VEP (Proix et al., 2017;V. K. Jirsa et al., 2017), many studies have developed, improved, and validated the approach. From a methodological point of view, the VEP has been specified as a fully Bayesian probabilistic generative model (Hashemi et al., 2020), in which the importance of informative*a priori*knowledge has been highlighted (Hashemi et al., 2021). VEP has been validated with synthetic data and applied to empirical SEEG recordings for the estimation of the EZN with different inference techniques: maximum a posteriori (Vattikonda et al., 2021), Hamiltonian Monte Carlo (Jha et al., 2022), and simulation-based inference (Hashemi et al., 2023). From a clinical perspective, the performance of the VEP has been recently assessed retrospectively in a cohort of 53 patients by comparing the predictions of the VEP with the predictions of clinical experts, with respect to the surgical outcome of the patients (Makhalova et al., 2022;Wang et al., 2023). One essential conclusion was that the concordance of the predictions was better for seizure-free compared to non-seizure-free patients. In addition, it is worth mentioning that the VEP methodology we are considering in the present study to identify EZN is currently at the heart of an ongoing large prospective multi-centric clinical trial (EPINOV, NCT03643016;V. Jirsa et al., 2023), with more than 300 patients, in order to evaluate its abilities to help plan surgery strategies.

### 1.4 Objective of the study

All the previously mentioned studies have used NMM to estimate the EZN from empirical observations. Indeed, considering the high spatial resolution of NFM, using NFM for model inversion is still a very difficult task (Vattikonda et al., 2023). For validation, that is, comparing the parameters estimated from synthetic data to the parameters used to simulate the same synthetic data (ground truth), all these studies have simulated synthetic observations with NMM. In other words, these studies have used only NMM for both simulation and estimation.

As emphasized previously, the repertoire of spatiotemporal dynamics generated with NFM is significantly more extended than that of NMM. Indeed, some specific patterns observed in empirical data, such as traveling waves, cannot be explained with NMM.

To push further the validation process of NMM for the estimation of the EZN, and demonstrate the relevance of its usage in a clinical context, it is therefore necessary to simulate synthetic observations with high spatial resolution NFM. This procedure has already been proposed inWang et al. (2023)and the authors demonstrated its proof of concept for one dataset. In the present study, we carried out an extensive and systematic evaluation of this approach in order to address two fundamental questions: what is the performance of the neural mass-based VEP pipeline when the ground truth simulated with NFM is known? And under which conditions would it succeed or fail?

### 1.5 Scheme of the study

In this study, we evaluated the performances of VEP with 14 patients with epilepsy. For each patient, we created a series of spatial configurations (ground truth), each defined with different EZN and PZN. We also varied the regions involved in these networks in terms of their size and their number. To disentangle the effects of using a simplified forward solution from the effects of absence of local coupling, two modeling features that an NMM inversion is intrinsically lacking, we simulated data in different conditions: with an NMM (control condition), with an NFM with local coupling equal to 0 (to evaluate the effects of forward model only), and with an NFM with local coupling equal to 2 and 4 (to evaluate the effects of local coupling only). Then, for each simulation, the EZN was systematically estimated with an NMM equipped with the reduced 2D Epileptor. Predictions were evaluated at the individual and group level in terms of goodness of fit and classification metrics. Finally, we separately demonstrated the effects of the two key limitations of the NMM-based estimation, namely the simplified forward solution and the absence of local propagation, with two specific examples.

## 2 Materials and Methods

### 2.1 Selection of patients and data acquisition

We selected fourteen patients suffering from drug-resistant focal epilepsy who underwent a standard presurgical protocol at La Timone Hospital in Marseille. Informed written consent was obtained for all patients, and the study was approved by the local ethics committee (Comité de Protection des Personnes sud Méditerranée 1).

The evaluation included non-invasive T1-weighted (T1W) MRI (magnetization prepared rapid acquisition gradient echo sequence, either with repetition time = 1.9 s, echo time = 2.21 ms (1 patient, id010 inSupplementary Table 1) or repetition time = 2.3 s, echo time = 2.98 ms (13 patients), voxel size 1.0 × 1.0 × 1.0 mm) and diffusion-weighted (DW) MRI (diffusion tensor imaging-MR sequence, either with angular gradient set of 64 directions, repetition time = 10.7 s, echo time = 95 ms, voxel size 1.95 × 1.95 × 2.0 mm, b-weighting of 1000 s/mm^2^(2 patients, id010 and id022 inSupplementary Table 1), or with angular gradient set of 200 directions, repetition time = 3 s, echo time = 88 ms, voxel size 2.0 × 2.0 × 2.0 mm, b-weighting of 1800 s/mm^2^(12 patients)). Images were acquired using a Siemens Magnetom Verio 3T scanner.

SEEG electrodes (10–18 contacts 2 mm long and separated by 1.5 mm for all patients) were implanted individually for each patient as part of the clinical routine and according to the hypotheses about the EZN. A post-implantation computed tomography (CT) scan was performed to obtain the location of the implanted electrodes.

These patients were selected so as a majority of regions from each of the four cortical lobes of the brain (frontal, parietal, occipital, and temporal) could be represented. We considered that it was unnecessary to include more patients, especially if the clinical hypothesis about the localization of their EZN (and the related implantation) was similar to the ones already present in the study.

### 2.2 General workflow

**Fig. 1.**
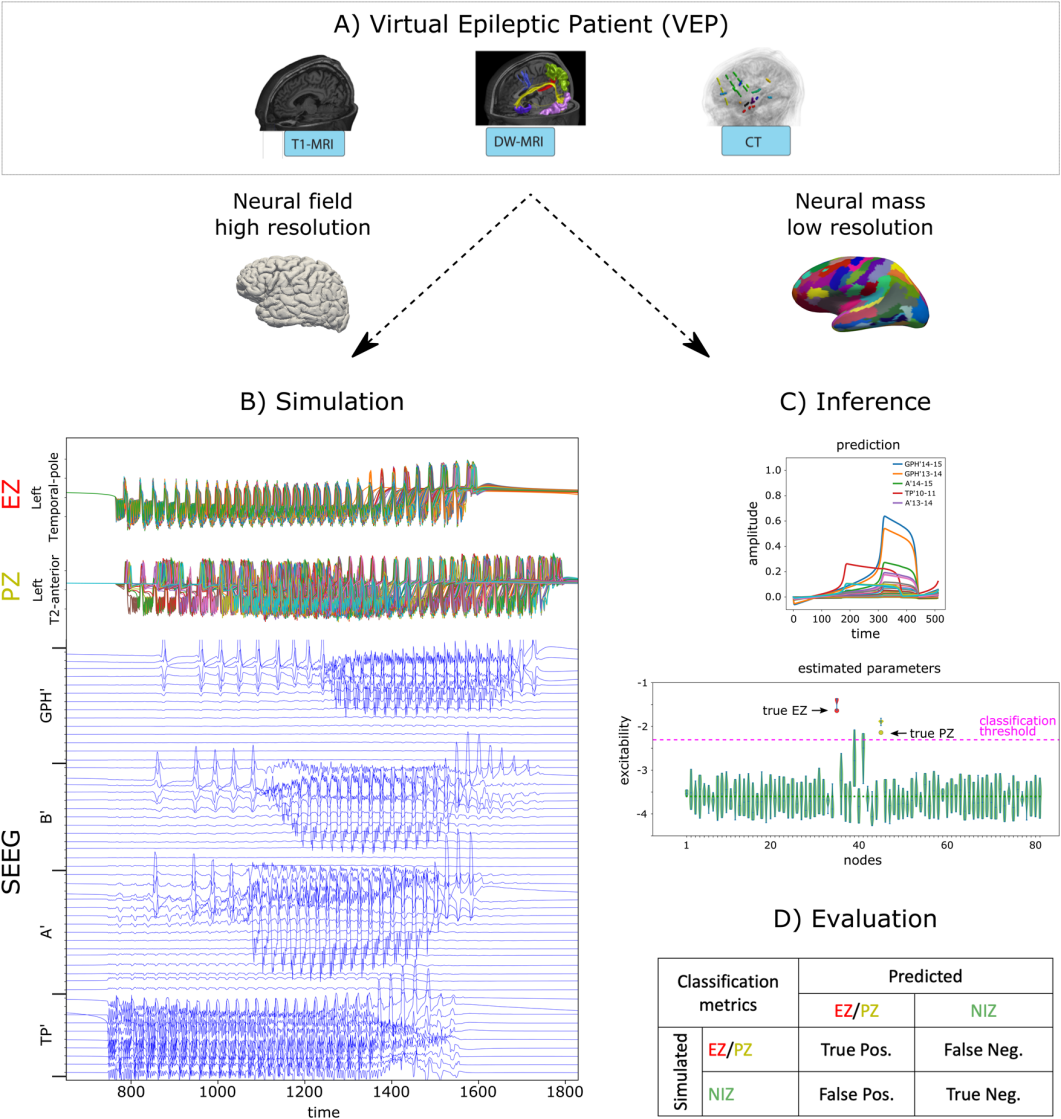
General workflow of the study. (A) Structural neuroimaging data of the patient are used to build a personalized brain network model: the Virtual Epileptic Patient (VEP). In particular, the T1-MRI, the DW-MRI, and the CT scan specify the brain space, the long-range connectome, and the position of the SEEG contacts respectively, which are essential attributes of a personalized VEP. (B) Using a specific spatial configuration of the Epileptogenic and Propagation Zone Networks (EZN and PZN respectively) and a high spatial resolution Neural Field Model (NFM) composed of 81924 cortical and subcortical nodes, synthetic data are simulated at the brain level for all the nodes (top panel: EZN and PZN nodes are shown on the first and second row respectively) and at the SEEG level (bottom panel). (C) Data features are extracted from the SEEG time series, and a low spatial resolution Neural Mass Model (NMM) composed of 162 nodes is used to predict the epileptogenicity (or excitability) of each node. (D) The estimation of the EZN and PZN is evaluated by comparing the ground truth excitabilities used to simulate the data, with the predicted ones (NIZ: Non-Involved Zones).

This section summarizes the pipeline used in the present study (Fig. 1). Specific details regarding the modeling, the creation of the synthetic dataset, and the estimation of the EZN with NMM and its evaluation are provided in subsequent sections.

First, a common procedure was applied to build an individualized VEP for each patient (Fig. 1A). A brain segmentation was computed from the T1W MRI with the Freesurfer software package (http://surfer.nmr.mgh.harvard.edu) and parcellated with respect to the VEP atlas (Wang et al., 2021). We used the ico6 spatial resolution, providing a cortical surface composed of 81924 vertices (~1.5 mm between the vertices). The structural connectome was derived from the DW MRI with the MRtrix software package (https://www.mrtrix.org) and the structural connectivity matrix (SC), corresponding to long-range (or global) coupling, was computed by counting the streamlines connecting the parcels of the VEP atlas (Vattikonda et al., 2021;Wang et al., 2023). The location of each contact of the SEEG electrodes was obtained from the CT scan using GARDEL (Medina Villalon et al., 2018) and coregistered to the MRI space. Together with the brain surfaces and the SC, these elements constitute the anatomical structure of an individualized VEP and constrain the spatiotemporal dynamics of the neuronal activity generated within the brain of the patient and measured with the SEEG electrodes. Second, we built a series of {EZN, PZN} spatial configurations based on individual clinical hypotheses. Clinical hypotheses (Supplementary Table 1) contain the list of regions suspected to be part of EZN or PZN. They were provided by the clinicians of La Timone Hospital in Marseille based on the presurgical evaluation of each patient, including the computation of an epileptogenicity index for each brain region (Bartolomei et al., 2008).

Then, we simulated each configuration in 4 different conditions (Fig. 1B): with an NMM and with an NFM for increasing strengths of the local coupling (lc): 0, 2, and 4. The 4 conditions were simulated using the full 5D Epileptor and labeled respectively lc_none, lc_0, lc_2, and lc_4. Increasing the strength of local coupling increases the speed of propagation of brain activity (Proix et al., 2018). It makes regions seize earlier (Fig. 3B, C and D, first column: the onset time of contact GC6-7 in simulated data), but may also change the number of recruited regions. This range of local coupling strengths also needs to maintain a balance with the other parameters, such as structural connectivity or global coupling (Spiegler & Jirsa, 2013). Indeed, for higher values of local coupling strength, the propagation of activity gets dominated by local coupling (fast propagation) and regions in PZN seize immediately after regions in EZN: it becomes almost impossible to distinguish EZN from PZN. Finally, simulated brain signals were multiplied by a gain matrix to form the measurements at the SEEG contacts, that is, the forward solution. Then, for each synthetic SEEG dataset (the observations), we used a probabilistic inversion scheme based on an NMM equipped with the reduced 2D Epileptor (Fig. 1C). This automatic procedure consists in finding the most plausible values (maximum a posteriori,Section 2.5.2) for each of the free parameters of the NMM of the patient such that the predictions of the model best fit the observations.

The quality of the estimation was evaluated by two ways (Fig. 1D). We computed a goodness of fit to evaluate how well the SEEG signals predicted by the statistical model fitted the observations. Based on the estimated parameters of the model, we also derived an estimation of the {EZN, PZN} that we compared to the ground truth {EZN, PZN} used to simulate the data by means of classification metrics (precision, recall, F1 score).

### 2.3 Modeling brain activity

#### 2.3.1 Neural field model

In this section, we describe the NFM brain network model used to simulate synthetic data for the conditions lc_0, lc_2 and lc_4.

##### 2.3.1.1 Simulating brain activity

We used a mixed approach to represent the brain space, namely an NFM for the cortical structures and an NMM for the subcortical structures.

One reason to justify the importance of using NFM when modeling SEEG signals is the possibility to compute an accurate forward solution when the orientation of each source (with respect to the SEEG electrodes) is well defined. This is indeed the case in cortical structures where neuronal populations are oriented perpendicular to the surface and each node (vertex) can expressitsown orientation. However, there is so far no consensus in the literature about such a systematic orientation property in the subcortical structures, and therefore, no proper way to implement an accurate forward solution. Because of this limitation, subcortical structures were modeled with NMM and their associated forward solution (Section 2.3.2). Nevertheless, the fact that in this study, we restricted the location of EZN and PZN to cortical structures mitigates the effects of this simplification.

In this context, the brain space was defined with a high spatial resolution for the cortical surface (81924 nodes) and a low spatial resolution for the subcortical structures (18 nodes).

The full five-dimensional Epileptor neuronal model was assigned to each node and activity of$node_{i}$located at$x_{i}$, with1≤i≤81924+18, was given by the following equations (Proix et al., 2018):

$$\begin{array}{l} \;\;\;\;\;{\dot{u}}_{1,i}=u_{2,1}-f_{1}(u_{1,i},q_{1,i})-\nu_{i}+I_{1}+LC_{scaling}\gamma_{11}w_{1}*S(u_{1,i},\;\theta_{11})\\ \;\;\;\;\;\;\;\;\;\;\;\;\;\;\;\;\;\;+GC_{scaling}\sum_{j=1}^{162}C_{i,j}S({\tilde{u}}_{1,j},\;\theta_{GC})\\ \;\;\;\;{\dot{u}}_{2,i}=1-5u_{1,i}^{2}-u_{2,i}\\ \;\;\;\;{\dot{v}}_{i}=\frac{1}{\tau_{0}}(4(u_{1,i}-u_{0}(x_{i}))-v_{i})\\ \;\;\;\;\;{\dot{q}}_{1,i}=-q_{2,i}+q_{1,i}-q_{1,i}^{3}+I_{2}+0.002g(u_{1,i})\\ \;\;\;\;\;\;\;\;\;\;\;\;\;\;\;-0.3(v_{i}-3.5)+LC_{scaling}\gamma_{22}w_{2}*S(q_{1,i},\theta_{22})\\ \;\;\;\;\;\;\;{\dot{q}}_{2,i}=\frac{1}{\tau_{2}}(-q_{2,i}+f_{2}(q_{1,i}))\\ with\\ \;\;\;\;g(u_{1,i})=\int_{t_{0}}^{t}e^{-(t-s)/\tau_{12}}(a_{12}u_{1,i}+LC_{scaling}\gamma_{12}w_{12}*S(u_{1,i},\theta_{12}))ds\\ \;f_{1}(u_{1,i},q_{1,i})=\{\begin{array}{c} u_{1,i}^{3}-3u_{1,i}^{2}\;\;\;\;\;\;\;\;\;\;\;\;\;\;\;\;\;\;\;\;\;\;\;\;\;\;\;\;\;if\;u_{1,i}<0\\ (q_{1,i}-0.6{\left(v_{i}-4\right)}^{2})u_{1,i}\;\;if\;u_{1,i}\geq 0 \end{array}\\ \;\;\;f_{2}(q_{1,i})=\{\begin{array}{c} 0\;\;\;\;\;\;\;\;\;\;\;\;\;\;\;\;\;\;\;\;\;\;\;\;\;\;\;\;\;\;if\;q_{1,i}<-0.25\\ 6(q_{1,i}+0.25)\;\;if\;q_{1,i}\geq -0.25 \end{array} \end{array}$$

with$I_{1}=3.1$,$I_{2}=0.8$,$\gamma_{11}=0.17$,$\gamma_{22}=0.32$,$\gamma_{12}=0.03$,$\theta_{11}=-1$,$\theta_{GC}=-1$,$\theta_{22}=-0.5$,$\theta_{12}=-1$,$\tau_{0}=2857/2$,$\tau_{2}=10$,$\tau_{12}=100$,$a_{12}=3$. Here, all parameters and variables have arbitrary units. Briefly, this phenomenological model couples together three populations,$u_{i}$,$v_{i}$and$q_{i}$, evolving at different time scales. With a slow time scale, the population$v_{i}$, usually referred as the slow permittivity variable, controls when the system enters and leaves the ictal state, which crucially depends on the fixed excitability$u_{0}\left(x_{i}\right)$of$node_{i}$, that is, its aptitude to autonomously generate seizure without any input from other nodes of the network. The popations with fast and intermediate time scales,$u_{i}$and$q_{i}$, account respectively for the presence of (low-voltage) fast oscillations and spike-wave discharges during the ictal state. Default values of the parameters have been chosen to best mimic characteristic features of spontaneous seizures recorded in patients with epilepsy (and particularly the presence of fast oscillations and spike-wave discharges;V. K. Jirsa et al., 2014), as well as the transitions between interictal and ictal activity (direct current shift at seizure onset, slowing down of activity at seizure offset). The observable activity of one Epileptor$node_{i}$is the quantity$q_{1,i}-u_{1,i}$which represents the cooccurence of spike-wave discharges with fast oscillations.

_$LC_{scaling}$_and$GC_{scaling}$are the coefficients used to scale local and global coupling at the whole network level, respectively. The local coupling termsw*S(u,θ)correspond to the spatial convolution at position$x_{i}$of a Laplacian kernelw(cutoff: 10 mm) with the local firing rate implemented with the Heaviside step function:S(u,θ)=0ifu<θand1otherwise.$C_{i,j}$is the connection strength between the region$node_{i}$is belonging to and regionjand${\tilde{u}}_{1,j}$is the averaged activity of regionjacross all nodes belonging to the region. For more details regarding the general mechanic of the neuronal model, the technical implementation of the coupled oscillators$u_{i}$and$q_{i}$, the value taken by each parameter, or the use of the model in the context of NFM, we refer the interested reader toV. K. Jirsa et al. (2014)andProix et al. (2018).

For subcortical nodes modeled as point sources NMM, the equations read the same except that the local connectivity is not considered ($\gamma_{11}=\gamma_{22}=\gamma_{12}=0$).

Whole-brain dynamics was integrated with a fourth-order Runge-Kutta deterministic integration scheme (step size: 0.1) for 20000 samples. In this study, we chose the millisecond as the unit of time and each simulation was therefore 2000 ms long. Initial conditions were set so that the simulated network could start in the interictal state and far away from seizure onset.

###### 2.3.1.1.1 Dynamics of the Epileptor model

Linear stability analysis of the Epileptor (a complete bifurcation analysis is proposed inEl Houssaini et al., 2020) has shown that depending on its excitability$u_{0}$, the system could exhibit different stability regimes, as described in previous studies (Proix et al., 2014;V. K. Jirsa et al., 2017) and used in previous simulation and inference investigations (Hashemi et al., 2020,^2021^;Jha et al., 2022;Vattikonda et al., 2021). For an excitability$u_{0}$lower than a critical value -2.05, and without any external input from other nodes of the network (no coupling), an isolated Epileptor has a single stable fixed point and does not seize autonomously. When the excitability equals the critical value, a saddle-node bifurcation occurs and an unstable fixed point appears. Then, seizures happen spontaneously and the node is part of the Epileptogenic Zone Network (EZN). When excitability is lower but still sufficiently close to the critical value, a relatively small external input from the network may destabilize the system and trigger a seizure in the recruited node: the node is part of the Propagation Zone Network (PZN). Otherwise, if the excitability is lower and far enough from the critical value, the external input cannot trigger a seizure and the node is in the Non-Involved Zone (NIZ).

Importantly, this means that one single threshold to distinguish PZN nodes from NIZ nodes across the whole network does not exist in general. Instead, this threshold depends on the intrinsic properties of the node under consideration, the configuration of the network activity, and the coupling parameters (SC,$GC_{scaling}$and$LC_{scaling}$).

##### 2.3.1.2 Mapping brain activity to SEEG measurements

For cortical nodes, the gain from$node_{i}$to SEEG contactklocated respectively at positions${\text{x}}_{\text{i}}$(vertexi) and${\text{r}}_{\text{k}}$was computed by considering the brain as an infinite homogeneous volume conductor with the following equation (Sarvas, 1987):

$$g_{k,i}=\frac{a_{i}}{4\pi \sigma }\text{Q}*\frac{{\text{r}}_{\text{k}}-{\text{x}}_{\text{i}}}{|{\text{r}}_{\text{k}}-{\text{x}}_{\text{i}}{|}^{3}}$$

where$a_{i}$is the area of vertexi(one-third of the sum of surrounding triangles areas) and counterbalances the (relatively small) spatial inhomogeneities due to the surface discretization,σis the homogeneous electrical conductivity,Qis the unitary dipolar moment (the unit vector normal to the surface at vertexi), and$\text{Q}*\left({\text{r}}_{\text{k}}-{\text{x}}_{\text{i}}\right)$is the dot product ofQand the vector$\left({\text{r}}_{\text{k}}-{\text{x}}_{\text{i}}\right)$, going from vertex*i*to SEEG contact*k*. For subcortical nodes, the gain was computed using the NMM equation (Section 2.3.2).

SEEG measurements were computed with the following equation:

$$SEEG_{k}(t)=\sum_{i=1}^{81924+18}g_{k,i}(q_{1,i}(t)-u_{1,i}(t))$$

where$q_{1,\;i}-u_{1,\;i}$is the instantaneous observable activity of Epileptor$node_{i}$(Section 2.3.1.1).

#### 2.3.2 Neural mass model

In this section, we describe the NMM brain network model used to simulate synthetic data for the condition lc_none.

##### 2.3.2.1 Simulating brain activity

The point source network model was composed of 162 nodes (cortical: 146, subcortical: 18). The equations read the same as the equations of NFM (Section 2.3.1.1) except that1≤i≤162, no local connectivity is considered ($LC_{scaling}=0$) and the averaged activity${\tilde{u}}_{1,j}$of region*j*is the activity of its single node$u_{1,j}$:${\tilde{u}}_{1,j}=u_{1,j}$.

##### 2.3.2.2 Mapping brain activity to SEEG measurements

The gain from$node_{i}$to SEEG contactklocated respectively at positions${\text{x}}_{\text{i}}$(vertexi) and${\text{r}}_{\text{k}}$was computed with the following equation:

$$g_{k,i}=\frac{1}{4\pi \sigma }\sum_{j=1}^{N_{i}}\frac{a_{j}}{|r_{k}-x_{j}{|}^{2}}$$

whereσis the homogeneous electrical conductivity, andjidentifies one of the$N_{i}$vertices belonging to regioniand$a_{j}$is its area (Section 2.3.1.2).

Unlike NFM nodes, NMM regions cannot be properly represented by electrical dipoles with well-defined fixed position and orientation, due to their macroscopic sizes (Section 1.2). Instead, NMM systematically assumes orientations which maximize the gain values. Indeed, in the previous NFM gain equation (Section 2.3.1.2), if we assume that the dipolar momentQof$node_{i}$is systematically pointing into the direction of SEEG contactk(thus completely ignoring the curvature of the cortex and the normal to the surface at vertexi), then gain$g_{k,i}$is maximal, dot product$\text{Q}*\left({\text{r}}_{\text{k}}-{\text{x}}_{\text{i}}\right)$reads$\left|{\text{r}}_{\text{k}}-{\text{x}}_{\text{i}}\right|$, and we obtain the NMM gain equation.

SEEG measurements were computed with the following equation:

$$SEEG_{k}(t)=\sum_{i=1}^{162}g_{k,i}(q_{1,i}(t)-u_{1,i}(t))$$

### 2.4 Configurations

For each of the 14 patients, we defined a series of {EZN/PZN} spatial configurations following the same procedure. Each configuration defined the set of regions belonging to the EZN and to the PZN, based on the clinical hypothesis related to their specific EZN (Supplementary Table 1). Here, regions refer to the parcels of the VEP anatomical atlas (Wang et al., 2021), as stated inSection 1.2. We introduced 3 types of spatial configurations with increasing level of complexity. For the first type, only one region was included in the EZN. This resulted in a total of 62 configurations (corresponding to the 62 anatomical regions listed inSupplementary Table 1). In addition, to test the sensitivity of the estimation of the EZN with respect to the size of the region, we also considered a second series of configurations for which the size of each region was reduced to half. For this purpose, we computed the barycenter of each region. Starting from this cortical vertex, we used a region growing approach, successively adding surrounding vertices until the surface of the growing region reached half size of the surface of the full region. This procedure was followed by visual inspection. For the second type, one configuration was created for each patient in which all the regions from the hypothesis were included in the EZN (11 configurations, with at least 2 regions in the EZN, average number of regions in the EZN is 5.3 and maximum is 8). For the third type, one configuration was created for each patient for which the EZN contained one region and the PZN contained at least one region. Regions from the PZN were adjacent to the region of the EZN (11 configurations: 8 configurations with one region in PZN and 3 configurations with 2 regions in PZN). For each configuration, an excitability$u_{0}\left(x_{i}\right)$was set at each node$x_{i}$of the network to reflect the epileptogenicity of the node, that is, its ability to trigger a seizure. In this study, considering the dynamics of the Epileptor model (Section 2.3.1.1), we set the following excitability values: -1.6 for the EZN (the node can seize in an autonomous way), -2.1 for the PZN (the node needs input from the network to be recruited and to seize), and -3.6 for the NIZ (the node does not seize). These values have been determined from the analysis of the Epileptor model dynamics (Section 2.3.1.1).

Each spatial configuration was then simulated in 4 conditions: with an NMM and with an NFM using 3 different scalings of the local coupling$LC_{scaling}$(0, 2, and 4). These 4 conditions were simulated with the full five-dimensional Epileptor and in the following, they are identified as lc_none, lc_0, lc_2, and lc_4. The first condition lc_none corresponds to the best-case scenario, that is, a control condition in which an NMM is used for both the simulation and the estimation of the EZN. To dissociate the effects of the forward solution from the effects of the local coupling, we introduced the 2^nd^condition lc_0 which differs from NMM only by the way the forward solution is computed. Indeed, without local coupling, all the nodes belonging to the same region share the same dynamics (because of common initial conditions and a deterministic integration) which is also the same as the corresponding node from the 1^st^condition. And 3^rd^and 4^th^conditions added the effects of the local coupling.

For all configurations with empty PZN, the global coupling$GC_{scaling}$was set to 1 whereas for configurations with non-empty PZN, the global coupling was adjusted such that the propagation was sufficient to trigger a seizure in the PZN. Importantly, for each simulation, we checked that parameters, and especially the excitabilities, were consistent with the expected behavior (EZN, PZN, or NIZ) of each node (Section 2.3.1.1). Specifically, we verified that without any coupling, only EZN nodes seized and with coupling, only EZN and PZN nodes seized.Table 1summarizes the ensemble of simulated dataset, andFigure 2illustrates one simulation with an NFM using EZN = {Right-T2-Anterior}, PZN = {Right-T2-posterior},$GC_{scaling}$= 6 and$LC_{scaling}$= 2.

**Table 1.** Description of the synthetic dataset.

Spatial configurations	Count	Neuronal model and local coupling (lc)	Global coupling
EZN = {1 region}, PZN = {}	62	• NMM (lc_none) • NFM with lc = 0 (lc_0) • NFM with lc = 2 (lc_2) • NFM with lc = 4 (lc_4)	1.0
EZN = {1 half size region}, PZN = {}	62		
EZN = {multiple regions}, PZN = {}	11		
EZN = {1 region}, PZN = {1 or 2 regions}	11		adjusted

We defined 4 types of spatial configurations (1^st^column) to generate a set of spatial configurations across patients (2^nd^column). Each configuration was simulated in 4 conditions (3^rd^column) associated with different values for the local (3^rd^column) and global (4^th^column) coupling.

**Fig. 2.**
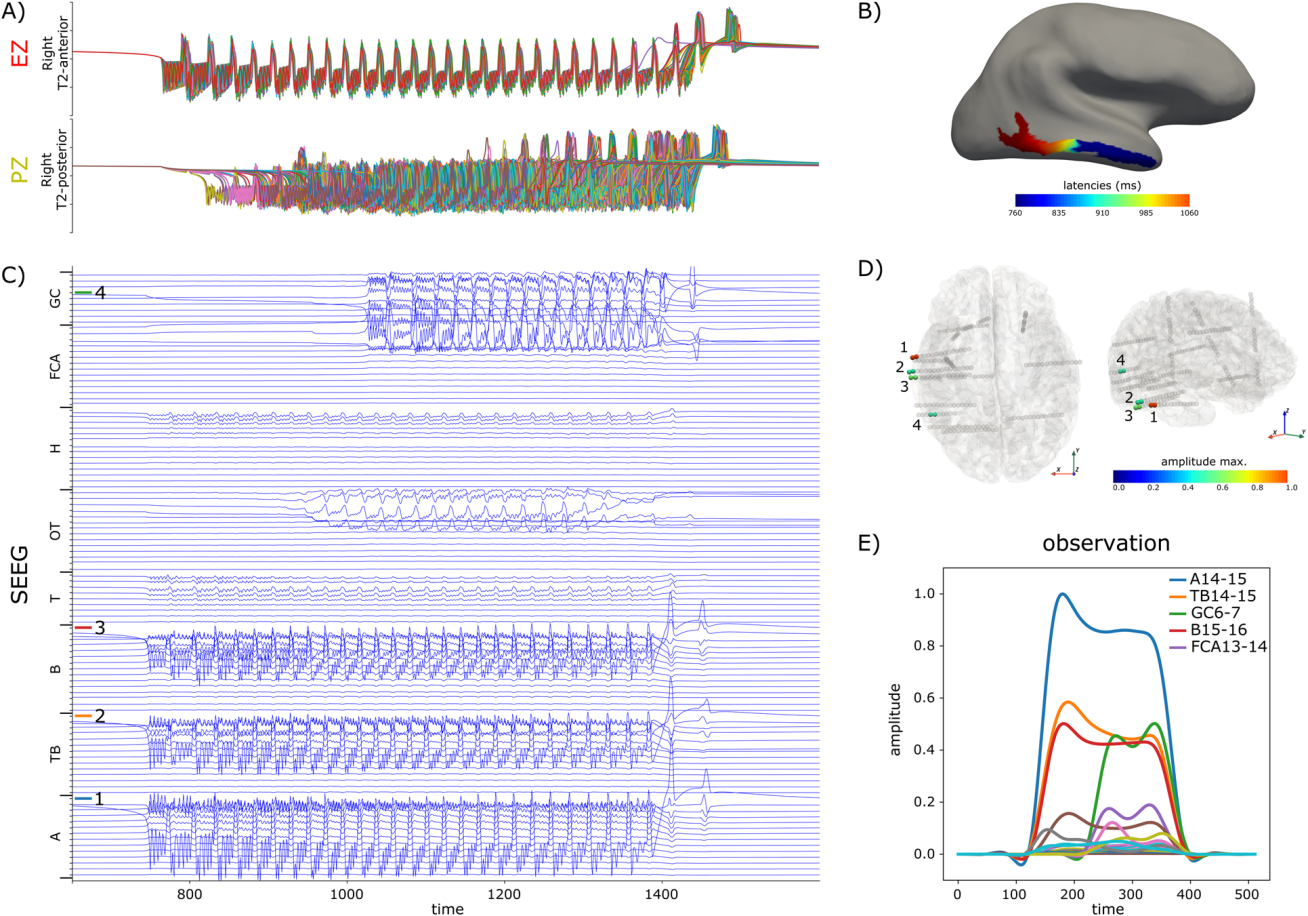
Synthetic data for patient id003. In this spatial configuration, EZN = {Right-T2-Anterior}, PZN = {Right-T2-posterior}. Local coupling strength is equal to 2. (A) Time courses of all vertices belonging to EZN (above) and PZN (below). (B) Spatial mapping of the onset latencies of the seizure. (C) Time courses of bipolar SEEG contacts. (D) SEEG implantation of the patient. (E) Data features (envelopes of high-frequency activity) computed from the SEEG bipolar contacts. The 4 bipolar contacts (1: A14-15, 2: TB14-15, 3: B15-16, 4: GC6-7) for which data features have the highest amplitude are highlighted in (C) and (D).

### 2.5 Estimation of the EZN

#### 2.5.1 Extraction of data features

The estimation of the EZN was not performed directly from the SEEG raw time series (Fig. 2C) but from their envelopes (Fig. 2E), which capture the amount of fast oscillations present in the signal simulated with a full five-dimensional Epileptor (Proix et al., 2017;V. K. Jirsa et al., 2017). This data feature extraction resulted in a set of observations matching the kind of predictions expected from an NMM equipped with a reduced 2D Epileptor. Indeed, with 2 state variables acting on different time scales, such a neuronal model cannot generate fast oscillations. The data features were computed separately for each SEEG bipolar contact similarly to previous studies (Hashemi et al., 2021;Vattikonda et al., 2021). The bipolar signal was high pass filtered at 50 Hz and squared. The envelope was computed with a moving average (window length = 50 ms), log-transformed, low pass filtered at 5 Hz, and normalized between 0 and 1. For the analysis, we only considered the time period during which ictal activity was present. Even if all simulated datasets were of same duration (2000 ms), the ictal period varied across dataset and we implemented a simple automatic procedure to detect the onset and the offset of each simulated seizure separately. It detected the 2 samples corresponding to the first up-crossing and last down-crossing ($t_{up}$and$t_{down}$) of a given threshold (here, 0.1). From the duration of this ictal interval,$d_{ictal}=t_{down}-t_{up}$, we only considered the extracted data features within the time interval$\left[t_{up}-d_{ictal}/2,t_{down}+d_{ictal}/2\right]$. The extracted signal was visually checked and downsampled to 512 time samples in order to avoid an potential bias during the fitting due to different length of the signal across conditions.

#### 2.5.2 Estimation

The probabilistic approach of the estimation procedure has already been extensively detailed in previous studies (Hashemi et al., 2020,^2021^,^2023^;Jha et al., 2022;Vattikonda et al., 2021;Wang et al., 2023). Given a set of observationsx(the data features described in previousSection 2.5.1) and using Bayes’ theorem, the posterior density of parametersθis given by:

p(θ|x)=p(θ)p(x|θ)/​p(x)

wherep(θ)is the prior distribution ofθ,p(x|θ)is the likelihood function, andp(x)is the marginal likelihood (independent ofθ). In this study, the parameters were inferred with a maximum a posteriori (MAP) approach which estimates the mode of the posterior density:

$$\theta_{MAP}\left(x\right)=\text{arg}\;\underset{\theta }{\max}p\left(\theta |x\right)$$

The computation of$\theta_{MAP}\left(x\right)$was performed in Stan’s probabilistic programming language (https://mc-stan.org) based on the iterative quasi-Newton optimization algorithm L-BFGS (Nocedal & Wright, 2006). At the first step, the parameters are initialized from their prior distribution using a random seed. Then at each step, the gradient of the posterior density is evaluated for the current parameters and used to update the parameters for the next step. Convergence is controlled by a set of tolerance values (sufficiently small norm of the gradient or sufficiently small change in posterior density or in parameter values). Because the estimation of MAP depends on the initial seed, L-BFGS algorithm was run 50 times, each time with a different seed. This procedure ended up with a set of$\theta_{MAP}\left(x\right)$which was then used to build the posterior marginal distributions of each of the parameters (described in the nextSection 2.6).

In order to evaluate the likelihood function (and its gradient), the inference process requires a generative model of brain activity, here a low spatial resolution NMM brain network model equipped with the 2D reduction of the Epileptor (Proix et al., 2014,^2017^). With the same notations as before, the equations read:

$$\begin{array}{l} {\dot{u}}_{1,i}=1-u_{1,i}^{3}-2u_{1,i}^{2}-v_{i}+I_{1}+GC_{scaling}\sum_{j=1}^{162}C_{i,j}S(u_{1,i},\theta_{GC})\\ \;\;\;\;\;\;\;={\dot{v}}_{i}=\frac{1}{\tau_{0}}(4(u_{1,i}-u_{0}(x_{i}))-v_{i}) \end{array}$$

Note that to be consistent with the implementation of NFM and NMM equipped with full 5D Epileptor described in previousSection 2.3, we used a global fast synaptic coupling acting on population$u_{1,i}$rather than a slow permittivity coupling acting on population$v_{i}$.

In this study, weakly informative priors were assigned to all free parameters of the model. In particular,$u_{0}\left(x_{i}\right)\sim N\left(-3.6,1\right)$for each node and$GC_{scaling}\sim N\left(1,1\right)$. To reduce the dimensionality of the parameter space and limit the complexity of the inference, initial conditions were not inferred and fixed to the values used for simulation.

### 2.6 Evaluation of performances

The estimation of the EZN was evaluated for each SEEG simulation with one measure of goodness of fit (GOF) and classification metrics. All these measures range from 0 to 1 (1 corresponds to a perfect fit).

The GOF measured the similarity between the observed and the predicted SEEG signals and was evaluated for each seed as the ratio of the explained variance (predictions) to the observed variance (equations in Supplementary Material). One GOF was computed for each of the 50 seeds, and only the optimizations for which the GOF was superior to the 3^rd^quartile of the 50 GOF were selected. From the selected optimizations, the GOF of the simulation was defined as the median of the GOF and the posterior distributions of each parameter were obtained from the normalized histogram of posterior modes (the area under a normalized histogram integrates to 1).

Classification aimed at attributing one class (either EZ or PZ or NIZ) to each node, given an estimation of its excitability (here, the median of the posterior marginal distribution). However, in the present context, there is no direct way to attribute a class to each node because no fixed boundary threshold based on excitability exists to differentiate PZ from NIZ nodes (Section 2.3.1.1). To circumvent this problem, we reduced the classification from 3 classes to 2 classes, either EZ_PZ or NIZ, and proposed a simple heuristic threshold to differentiate EZ_PZ from NIZ. Given such a heuristic classification threshold (explicitely defined thereafter), the general procedure to derive classification metrics followed three steps: 1) a class was assigned to each node: EZ_PZ if its estimated excitability was larger than the classification threshold, and NIZ otherwise; 2) considering NIZ as the null hypothesis, we compared for each node the estimated class with the true class and assigned either a type of error (false positive or false negative) or the kind of correct result (true positive or true negative); 3) we computed three classification metrics: precision (the fraction of nodes truly identified EZ_PZ among all nodes identified EZ_PZ), recall (the fraction of nodes truly identified EZ_PZ among all EZ_PZ nodes), and F1 score (harmonic mean of precision and recall). This general procedure was repeated for a series of classification thresholds spanning an extended range of values (from -5 to -1 with a step of 0.01). Finally, the classification threshold for which precision was maximal was selected, and the associated precision, recall, and F1 score were reported. This technique was applied independently for each simulation.

From a clinical perspective, the maximization of precision, equivalent to the minimization of false discovery rate, corresponds to a conservative choice if we consider estimated EZ_PZ regions as candidates for surgical resection.

Other classification approaches, particularly relevant in clinical context when ground truth is not known, have been proposed based on estimated excitabilities (Hashemi et al., 2020,^2021^,^2023^;Jha et al., 2022) or onset times extracted from predicted data features (Vattikonda et al., 2021,^2023^;Wang et al., 2023). Yet, these approaches eventually suffer from the choice of arbitrary thresholds to classify PZN. Here, because we are in a simulation study and the ground truth is known, we sidestep this problem by computing a threshold maximizing the precision. This classification bias in favor of precision but at the expense of recall is counterbalanced in the reported F1 score.

Also, for clarity and simplicity, we did not systematically distinguish EZ from PZ (NIZ is the null hypothesis) because we assumed that both EZ and PZ estimations would be globally affected the same way by the factors under consideration (forward model and local coupling). Of course, the situation would be different in a clinical context, where the distinction between EZN and PZN is essential, for instance to plan optimal surgical strategies based on seizure propagation control (Olmi et al., 2019).

## 3 Results

### 3.1 Examples

In this section, we illustrate key aspects of our results with two different spatial configurations of the EZN. These two didactic examples are illustrative of common phenomena observed repeatedly across simulations. For each configuration, and across the 4 simulated conditions (lc_none: NMM without local coupling, lc_0: NFM with no local coupling, lc_2 and lc_4: NFM with local coupling scaling equal to 2 and 4 respectively), we present the data features of the SEEG simulated observations, the predictions of the NMM (equipped with the reduced 2D Epileptor) with highest GOF, the GOF for the 50 optimizations (corresponding to 50 different random seeds of the optimization procedure,Section 2.5.2), and the posterior distributions of the excitability of the regions along with the ground truth of the configuration.

**Fig. 3.**
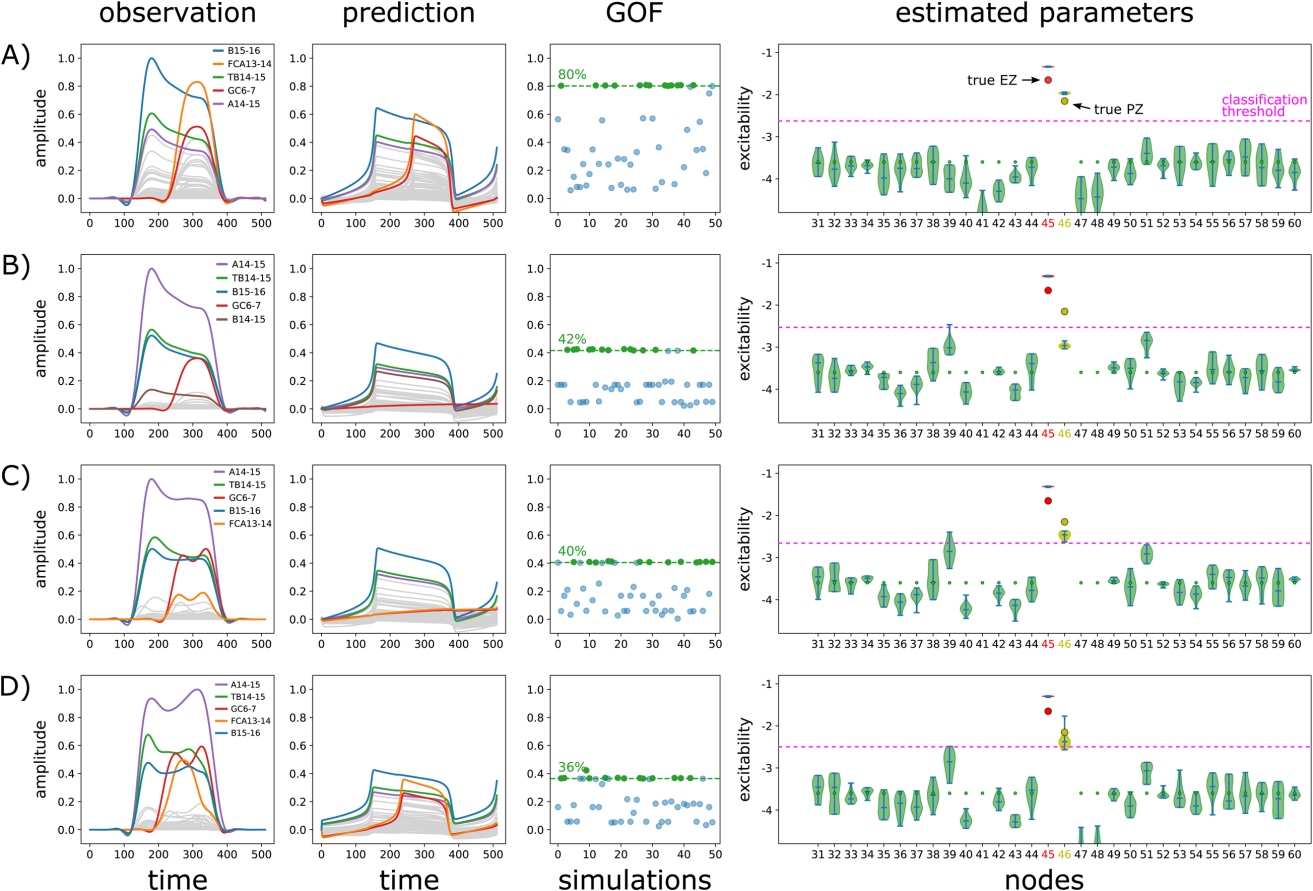
Estimation of EZN for patient id003 and EZN = {Right-T2-Anterior}, PZN = {Right-T2-posterior}. Each row corresponds to the brain network model used to simulate the data: (A) NMM (lc_none), (B) NFM with local coupling = 0 (lc_0), (C) NFM with local coupling = 2 (lc_2), and (D) NFM with local coupling = 4 (lc_4). The first column shows the data features extracted from the SEEG simulated observations. The second column shows the predictions of the NMM for the optimization seed with highest goodness of fit (GOF). In the legend, SEEG bipolar contacts are ordered according to their highest amplitude (colors are the same across rows). The third column shows the GOF of each optimization (50 seeds). The GOF is represented as a green dot if it is higher than the 3^rd^quartile, and in blue otherwise. The last column shows the posterior distributions of the excitability for a selection of nodes (regions). Excitability values used to simulate the data (ground truth) are represented as dots for each node. Colors highlight the ground truth of each node (red for EZN, yellow for PZN, green for NIZ). Please seeSupplementary Figure 1for the estimation of all the nodes from the right hemisphere. The synthetic raw data of condition C are illustrated inFigure 2.

The first example (Fig. 3) corresponds to patient id003 and EZN = {Right-T2-Anterior}, PZN = {Right-T2-posterior}. The synthetic raw data for the condition lc_2 are illustrated onFigure 2. Observed and predicted SEEG time courses are presented respectively on the first two columns (Fig. 3). In NMM simulated data (Fig. 3A, first column), we clearly see around time sample 100 the initiation of the seizure triggered in EZN (Fig. 3A, last column, node 45) and best captured by contact B15-16, followed around time sample 200 by the propagation of the seizure in PZN (Fig. 3A, last column, node 46), this time very well captured by contact FCA13-14. Considering now classification metrics (Fig. 3, last column), they can be measured visually by comparing across nodes the true excitability (represented as a dot) and the median of the posterior distribution (represented by the intermediate horizontal bar of each violin plot): the closer they are, the better the classification metrics. Once the classification threshold (represented as a pink horizontal dotted line) has been estimated (Section 2.6), all nodes for which the median of posterior distribution is higher than the threshold are positive: either true positives if they belong to EZN or PZN or false positives for those in NIZ. Precision is then the ratio of true positives among all positives, and recall is the ratio of true positives among all EZN or PZN nodes (sum of true positives and false negatives). In this example, the estimation of the EZN is very good in all 4 conditions with precision = 1 and recall = 1 except for the condition lc_0 (Fig. 3B, last column) for which recall = 0.5 (the posterior median excitability is higher in region 51 (NIZ) compared to region 46 (PZN)). However, considering the NFM simulations, two points are noteworthy. First, the underestimation of the excitability in PZN (region 46) is related to the data features themselves, which show a much higher quantity of signal (energy) recorded from EZN (Fig. 3B, first column: contacts A14-15, TB14-15, and B15-16) compared to PZN (Fig. 3B, first column: contact GC6-7). And second, excitability has been systematically overestimated in regions 39 (Right-T1-lateral-anterior) and 51 (Right-Collateral-sulcus), close to EZN but belonging to NIZ.

**Fig. 4.**
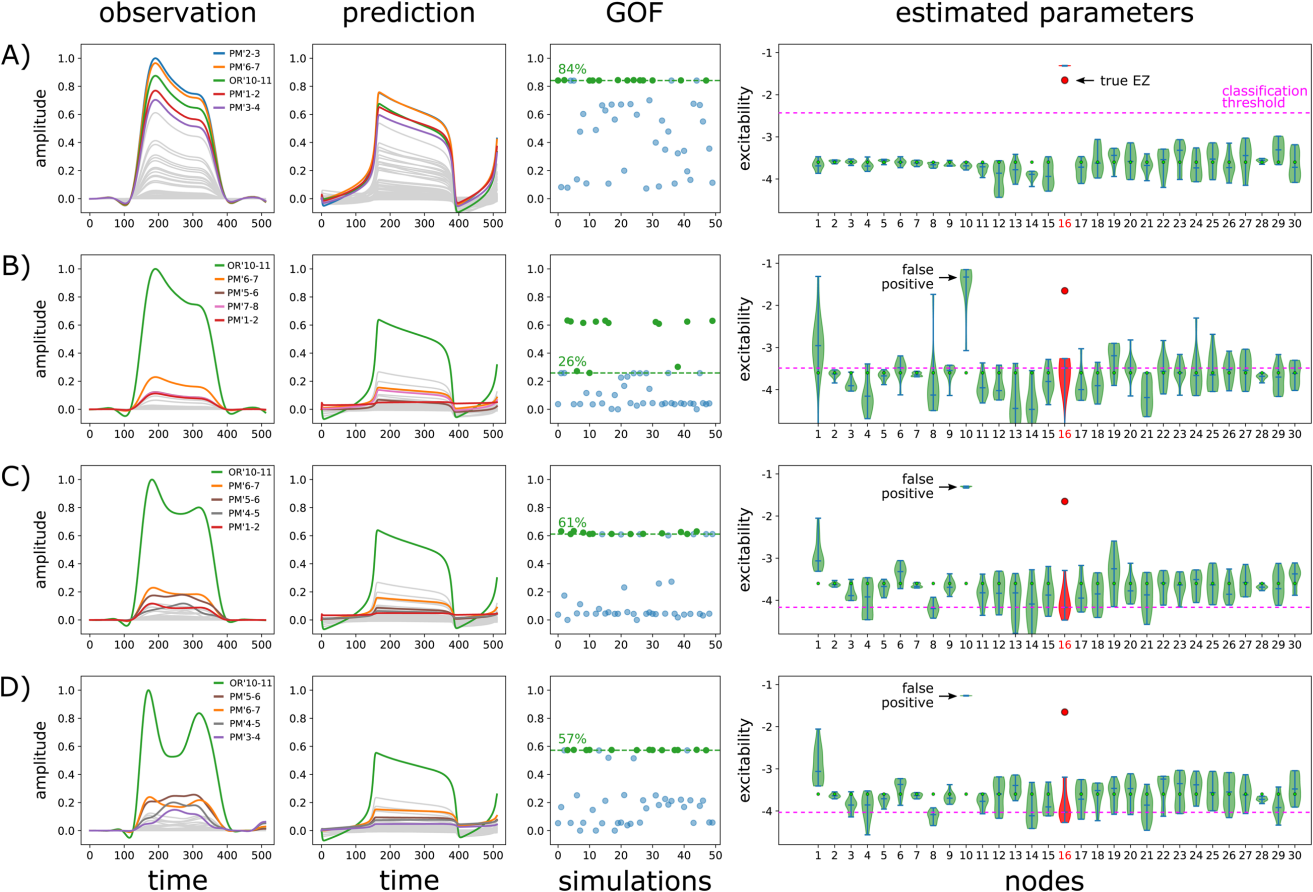
Estimation of EZN for patient id013 and EZN = {Left-F1-lateral-prefrontal}. Notations are the same as forFigure 3. Please seeSupplementary Figure 2for the estimation of all the nodes from the left hemisphere. (A) NMM (lc_none), (B) NFM with local coupling = 0 (lc_0), (C) NFM with local coupling = 2 (lc_2), and (D) NFM with local coupling = 4 (lc_4).

The second example (Fig. 4) corresponds to patient id013 with EZN = {Left-F1-lateral-prefrontal}, PZN = {}. While the estimation of the EZN is correct (region 16) in the control condition lc_none (Fig. 4A, last column), region 10 (Left-Middle-frontal-sulcus), which is spatially very close to region 16, is identified as being the EZN in data simulated with the NFM (Fig. 4B, C and D, last column). As in the previous example, this false positive can be explained by the characteristics of the data features which in this condition are particularly different between NMM and NFM, highlighting the important role of the gain matrix when computing a forward solution. Here, no significant difference is observed in the estimated parameters between conditions lc_0, lc_2, and lc_4 (Fig. 4B, C and D, last column), meaning that the effect of local coupling is limited.

We detailed these two didactic examples to present the main steps of our methodology and also to illustrate two phenomena observed recurrently across simulations. First, estimations from data simulated with NMM were good (Figs. 3Aand4A). And second, the differences observed between NMM and NFM simulated data, mostly due to the different forward models at work, could result in poor estimations of PZN (Fig. 3B) but also of EZN (Fig. 4B, C and D), depending on the spatial configuration.

### 3.2 Group results

**Fig. 5.**
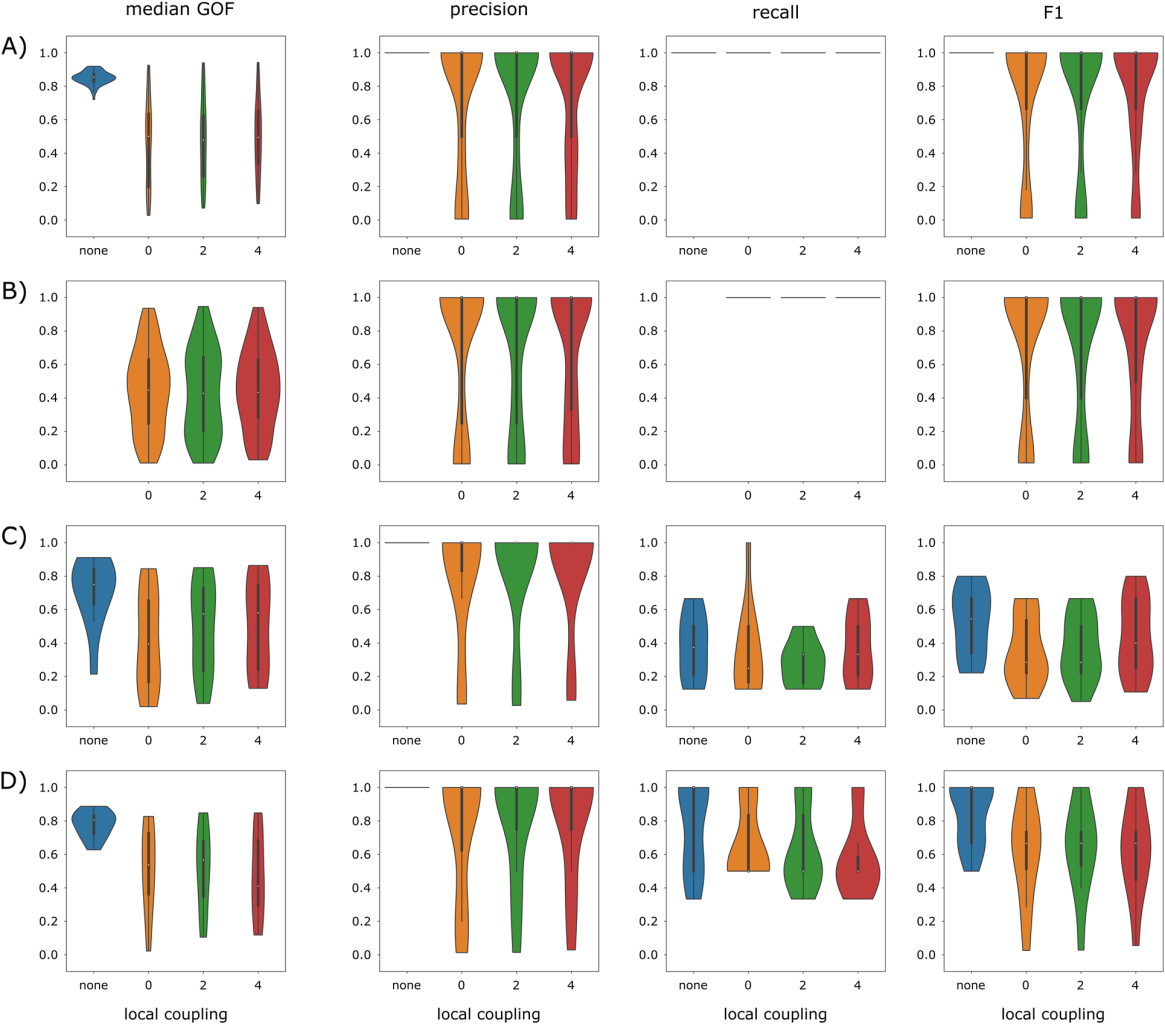
Evaluation of the estimation of EZN and PZN based on goodness of fit and classification metrics. Each line corresponds to one type of spatial configuration of the EZN. (A) EZN = {1 region}, PZN = {}, 62 simulations; (B) EZN = {1 half size region}, PZN = {}, 62 simulations; (C) EZN = {multiple regions}, PZN = {}, 11 simulations; and (D) EZN = {1 region}, PZN = {1 or 2 regions}, 11 simulations. Columns represent the median goodness of fit (GOF), the precision, the recall, and the F1 score respectively for each of the 4 conditions lc_none (blue), lc_0 (orange), lc_2 (green), and lc_4 (red). Each violin plot shows the distribution of a given measure across all simulations performed for one type of spatial configuration of the EZN and computed with a specific brain network model.

Distributions of GOF, precision, recall, and F1 score were computed across all simulations grouped by the 4 types of spatial configuration of the EZN and by the specific brain network model used to simulate the data: lc_none, lc_0, lc_2, and lc_4 (Fig. 5). Overall, regardless of the type of spatial configuration, the medians of the 4 evaluation metrics were systematically higher (or equal) for the control condition lc_none, compared to the 3 other conditions. The first type of spatial configuration (EZN = {1 region}, PZN = {},Fig. 5A) disclosed the most significant differences across the 4 conditions (Kruskal-Wallis H tests with p-value < 1e-06 for each metric). In particular, GOF was significantly higher for lc_none (median = 0.85) compared to the other conditions (median = 0.50, 0.48, and 0.49 for conditions lc_0, lc_2, and lc_4 respectively; Wilcoxon signed-rank tests with p-value < 1e-10 for each condition). Importantly, even if the difference was also significant for F1 score (Wilcoxon signed-rank tests with p-value < 2e-4 for each condition), the reproducible median value of 1 indicated a good estimation of the EZN for all conditions. Please note that for this configuration, because there is only one region to identify and precision is maximized, recall has to be 1 (this also applies to the next spatial configuration). For the second type of spatial configuration (EZN = {1 half size region}, PZN = {},Fig. 5B), performances were very similar and only slightly decreased (median GOF = 0.45, 0.43, and 0.43 for conditions lc_0, lc_2, and lc_4 respectively). For the third spatial configuration (EZN = {multiple regions}, PZN = {},Fig. 5C), the estimation of the EZN was degraded in all conditions as illustrated by the F1 score (median = 0.55, 0.29, 0.29, and 0.40 for each condition respectively). For the last configuration (EZN = {1 region}, PZN = {1 or 2 regions},Fig. 5D), the F1 scores indicated a better estimation of the EZN/PZN, compared to the previous configuration (median = 1.00, 0.67, 0.67, and 0.67 for each condition respectively). For the 3^rd^and 4^th^types of spatial configurations (Fig. 5C, D), the tendency of having a global decrease of performance for conditions lc_0, lc_2, and lc_4 compared to lc_none was conserved, but no longer significant (Kruskal-Wallis H tests with p-value = 0.1, 0.4, 0.6, and 0.2 for each metric of the 3^rd^configuration and p-value = 0.01, 0.3, 0.3, and 0.1 for each metric of the 4^th^configuration).

To summarize, we first observed that overall performances of the NMM inversion were good for data simulated with NMM and NFM for the 1^st^, 2^nd^, and 4^th^spatial configurations and worse for the more complex 3^rd^spatial configuration (for which an average of 5.3 regions were included in EZN/PZN). And second, the estimations based on NFM simulated data were systematically deteriorated compared to the estimations based on NMM simulated data. This tendency was similar across configurations, suggesting it was independent of the relative complexities of the spatial configurations. In addition, these results effectively disentangled the effects of using a simplified forward model from the effects of absence of local coupling during the NMM inversion. Comparing lc_none with lc_0 showed that the effects of the forward model were quite pronounced while comparing lc_0 with lc_2 and lc_4 showed that the effects of local coupling were limited (Fig. 5).

### 3.3 Effects of differences between NMM and NFM gain matrices

In this section, we studied the differences between the two very different approaches used by the NMM and NFM to compute the forward solution. For the NMM, the activity of a point-like region is projected to each SEEG contact by considering only a (weighted inversed squared) distance between the region and the SEEG contact (Section 2.3.2.2). For the NFM, the activity of each vertex is projected to an SEEG contact by also considering the orientation of the dipole at that vertex, with respect to the SEEG contact (Section 2.3.1.2).

In the previous section, we demonstrated with simulated data that the estimation of the EZN was significantly affected by the simplified gain matrix used by the NMM to compute the forward solution. In fact, the effects due to these different gain matrices are already present and visible in the synthetic SEEG data and the extracted data features. Also, as mentioned inSection 2.4, they were specifically pronounced when comparing conditions lc_none and lc_0. This is illustrated inFigure 6Awhere the sensitivity of the SEEG contacts to the activity of EZN and PZN differed between NMM and NFM (different gain matrices applied to an identical source activity): EZN and PZN activities are better detected on contacts B15-16 and FCA13-14 with NMM simulated data, and on contacts A14-15 and GC6-7 with NFM simulated data. Actually, a simple inspection of the gain matrices confirmed these differences. InFigure 6B, we compared the gains of the EZ (top panel) and PZ (bottom panel) regions for NMM (blue) and NFM (orange). For the NFM, these gains were obtained by summing the gains across all vertices belonging to the region of interest. This summation is justified by the fact that in the condition lc_0 (no local propagation and same initial conditions), all vertices of one region have the exact same activity. With this comparison, we confirmed the discrepancy that we observed in the data features: for NMM, the bipolar contacts most sensitive to the activity of the EZ and the PZ regions were B15-16 and FCA13-14 respectively, while for NFM, these bipolar contacts were A14-15 and GC6-7.

**Fig. 6.**
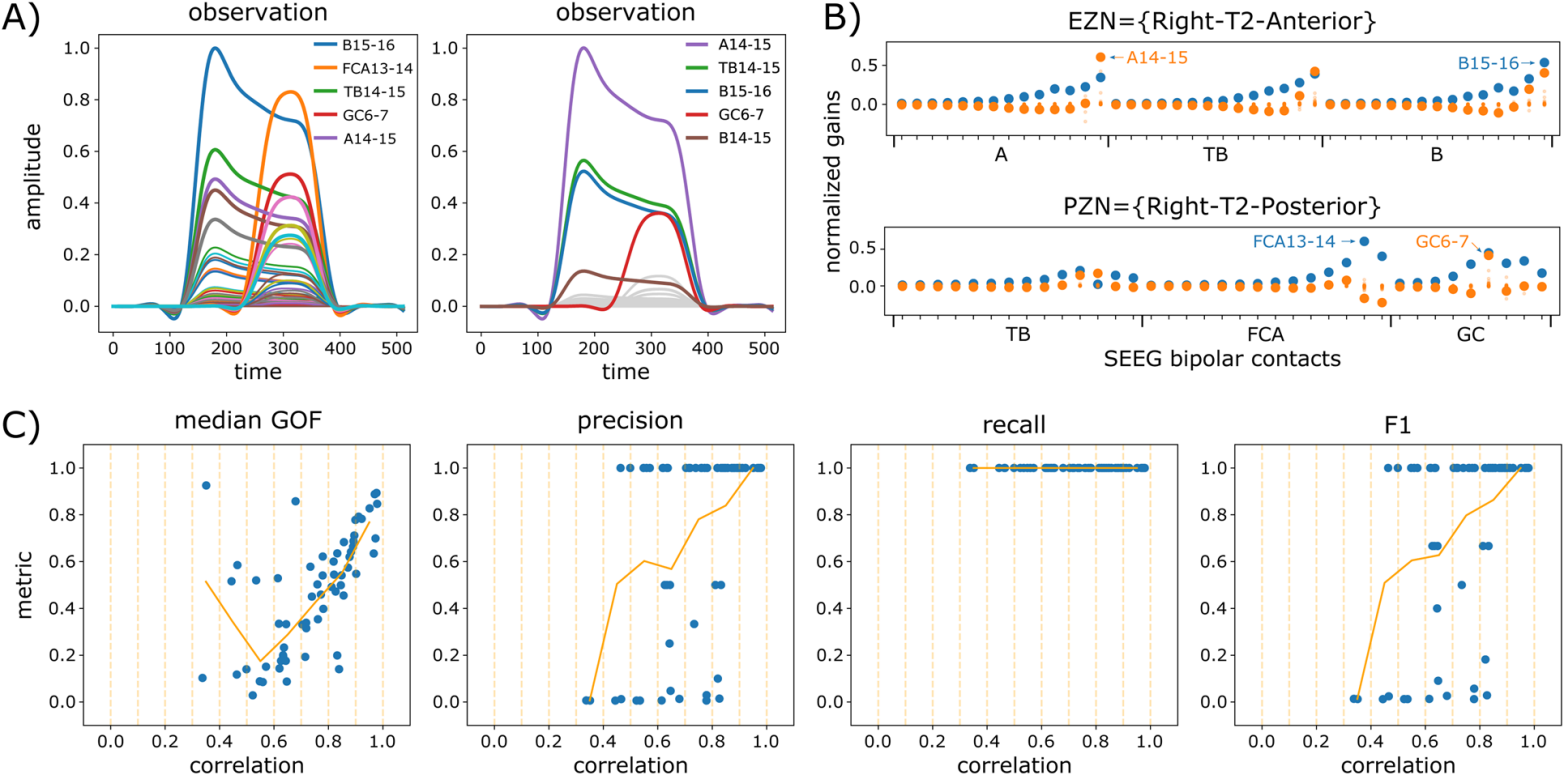
Differences between NMM and NFM gain matrices. (A) SEEG data features for patient id003 with EZN = {Right-T2-Anterior}, PZN = {Right-T2-posterior} in the condition lc_none (left), and lc_0 (right). (B) Normalized gains (vertical axis) of EZN = {Right-T2-Anterior} (top) and PZN = {Right-T2-posterior} (bottom) for SEEG bipolar contacts (horizontal axis) corresponding to NMM (blue) and to NFM (orange). (C) The four evaluation metrics (goodness of fit, precision, recall, and F1 score) plotted against the correlation between gains of NMM and gains of NFM. Each blue dot corresponds to one of the 62 simulations with EZN = {1 region} and PZN = {}, and for the condition lc_0. The orange curve represents the metric averaged over intervals of 0.1 of correlation.

We extended this comparison to the first set of 62 spatial configurations for which EZN = {1 region} and PZN = {} (Table 1) to assess whether the similarity of gain matrices could affect the estimation of the EZN (Fig. 6C). For each simulation, we computed a Pearson correlation coefficient between the NMM and NFM gains (normalized and rectified) for the EZ region. These coefficients ranged from 0.34 to 0.98 (all p-values for the test of zero correlation were inferior to 1e-5) and were pretty good predictors of the different evaluation measures (goodness of fit and classification metrics). In other words, the more similar the NMM and NFM gain matrices (for EZN), the better the estimation of the EZN (for condition lc_0). On the contrary, an EZN estimation located in a region where gain matrices really differ should be taken more cautiously.

### 3.4 Effects of local coupling

**Fig. 7.**
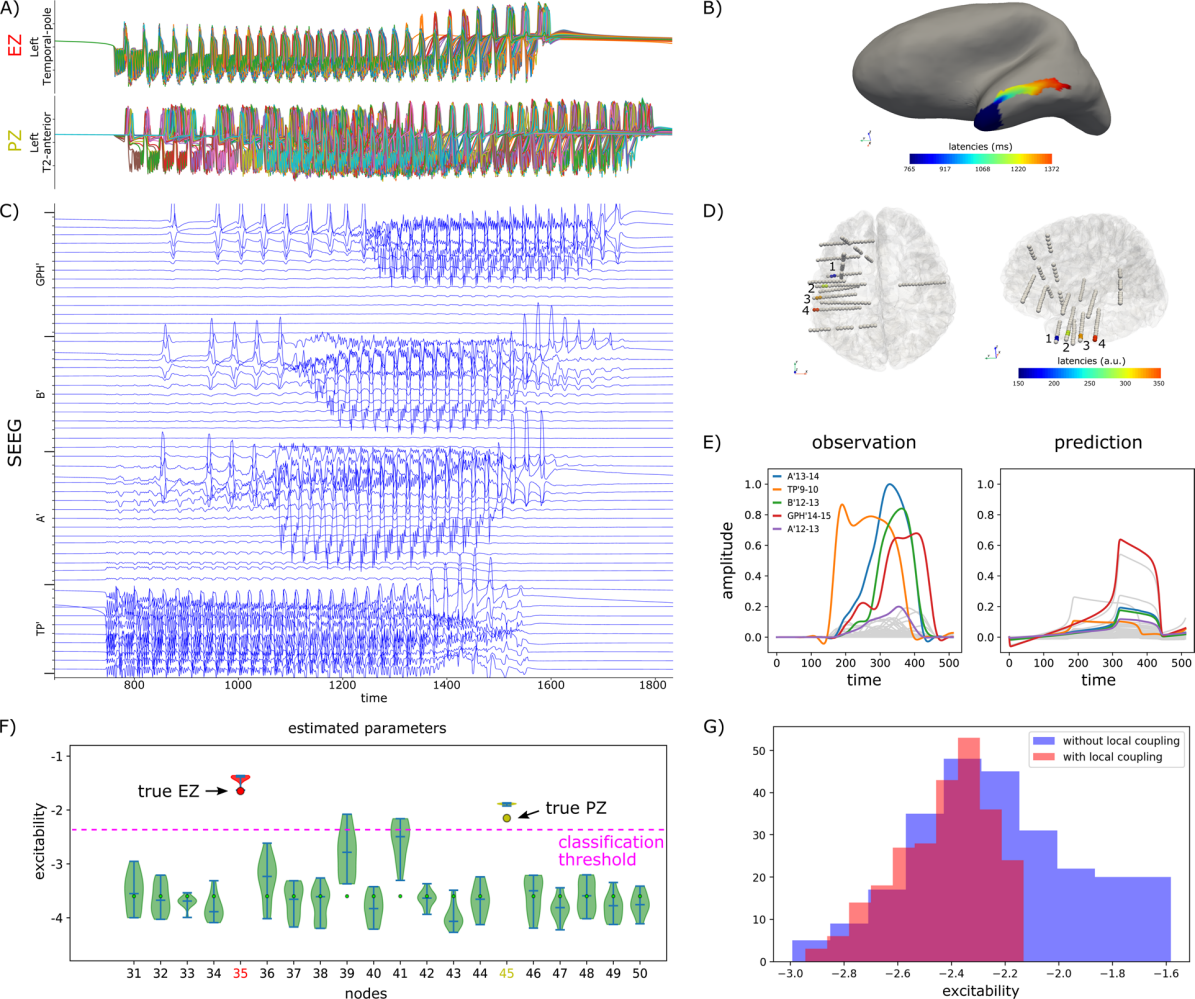
Simulation of seizure spread through local coupling. (A) Time courses of the vertices belonging to the EZN = {Left-Temporal-pole} (top row) and to the PZN = {Left-T2-anterior} (bottom row) simulated using an NFM with$GC_{scaling}=3$and$LC_{scaling}=4$. (B) Spatial mapping of the onset latencies of the seizure. The seizure starts in the blue region and propagates towards the red region. (C) Time courses of bipolar SEEG contacts. (D) SEEG implantation of the patient. Onset latencies are mapped with arbitrary units (a.u.) on bipolar contacts to show the propagation of the seizure (1: TP’9-10, 167 a.u.; 2: A’13-14, 270 a.u.; 3: B’12-13, 296 a.u.; 4: GPH’14-15, 320 a.u.). (E) Observed and predicted data features of the SEEG bipolar contacts. The 4 bipolar contacts for which observed data features show the highest amplitudes are highlighted in (D). (F) Estimation of the EZN. Notations are the same as forFigure 3. The EZN = {Left-Temporal-pole} and the PZN = {Left-T2-anterior} correspond to regions 35 and 45 respectively. Please seeSupplementary Figure 3for the estimation of all the nodes from the left hemisphere. (G) Estimation of the excitability of all vertices belonging to PZN with an NFM (Vattikonda et al., 2023) demonstrating a clear overestimation when local coupling is ignored.

In this section, we analyzed one additional simulation which emphasized the importance of local propagation in seizure spread and the necessity to consider it in the estimation of the EZN. In this simulation, we considered EZN = {Left-Temporal-pole} and PZN = {Left-T2-anterior},$GC_{scaling}=3$and$LC_{scaling}=4$(Fig. 7). The key difference with the previous simulations (the 11 spatial configurations for which the PZN was not empty,Table 1) is the cause of the seizure spread from EZN to PZN. In the previous simulations, the spread was mainly due to the global coupling between the EZN and the PZN. Indeed, we adjusted$GC_{scaling}$separately for each configuration such that even without local coupling (condition lc_0), the seizure effectively propagated from EZN to PZN and conditions lc_2 and lc_4 corresponded to a supplementary contribution of the local coupling, in addition to the global coupling. In the present simulation, the situation is very different because there is no seizure spread in absence of local coupling (even for higher values of$GC_{scaling}=3$), which reflects a low coupling strength between EZN and PZN coming from SC, and seizure propagates to PZN only when local coupling is introduced. The same procedure as previously described was used to infer the EZN and PZN in this simulation (Fig. 7). While the 2 regions were clearly identified among others, with a very good accuracy, it should still be noticed that posterior excitability values$u_{0}\left(x_{i}\right)$were overestimated (and higher than the critical value -2.05 for region 45 in PZN,Fig. 7F). Indeed, in the absence of global coupling between the EZN and the PZN, and without the possibility to propagate the seizure through local coupling, one hypothesis could be that the NMM used for the estimation of the EZN had no other way to explain the seizure in the PZN than to consider the region part of the EZN (the region could seize autonomously, without the effects of the network). A very recent study (Vattikonda et al., 2023) proposed a new approach for inferring the EZN using an NFM (instead of the NMM we used in the present study) which confirmed this hypothesis. By using the exact same simulation as the one presented in this section, the method demonstrated that as soon as the local coupling was considered in the estimation procedure, the excitability of the region part of the PZN was no longer overestimated (Fig. 7G).

## 4 Discussion

### 4.1 Summary

This study evaluated the estimation of an EZN in synthetic data with a low spatial resolution NMM, with a particular focus on disentangling the effects of using a simplified forward solution from the effects of absence of local coupling, two essential modeling features that the NMM approach is intrinsically lacking. For this purpose, we designed an ensemble of spatial configurations of the EZN, typical of what is observed in patients with epilepsy. We simulated these data with an NMM (control condition, lc_none) and with an NFM for increasing strengths of local coupling (lc_0, lc_2, and lc_4). This is to our knowledge the first time that data simulated with NFM are used for an extensive evaluation of inversion procedure based on NMM.

Our results indicated that, whatever the type of spatial configuration, the major difference was found between condition lc_none and other conditions (lc_0, lc_2, and lc_4). No real effect was observed between the latter conditions, emphasizing the general importance of the forward problem. For the simplest spatial configurations (Fig. 5A, B) for which the EZN was composed of one single region (full and half size), the effect of the forward problem was significant but still limited, with a median F1 score of 1 in all conditions. In such clearly identified clinical situations, it means that the estimation of EZN in empirical data using an NMM inversion can be considered as an efficient option. For more complex spatial configurations (Fig. 5C, D), a comparable tendency was present, not significant this time due to the lower number of configurations (11 < 62,Table 1), in addition to a general and non-negligible decrease of general performance for all conditions, especially more pronounced when the EZN was composed of multiple (not necessarily adjacent and eventually bilateral) regions (median F1 score = 0.55, 0.29, 0.29, and 0.40 for each condition respectively). For these more impacted configurations, which, moreover, correspond to clinically very relevant and more complex cases, inversion based on NMM should be interpreted more cautiously and deeper investigation may be necessary.

### 4.2 Effects of forward gain matrix

The drop of inference performance due to the gain matrix, which was identified in all spatial configurations of the EZN, was also unsurprisingly noticed in the extracted SEEG data features (Fig. 6A). Indeed, many studies already demonstrated the importance of using a high spatial resolution brain network approach, and the ensuing accurate forward gain matrix, for the modeling of SEEG activities. For instance inCosandier-Rimele et al. (2007a), the authors used a comparable model and found optimal parameters of two volume conductor models (infinite and 3-shell spherical head) to simulate a spike in the left middle temporal gyrus, and mimic an empirical one with high-fidelity. Interestingly, they characterized the attenuation of the electric potential with respect to the distance between the brain source and the SEEG contact and showed that for NFM, this attenuation (hyperbolic, 1/r) was less steep compared to NMM (parabolic, 1/r^2^). From this difference, they concluded that “neocortical sources of epileptic interictal activity have an extended nature that can be hardly represented by only one equivalent current dipole.” Another noteworthy consequence of using more realistic NFM (and associated gain matrix), and which needs to be considered for an accurate estimation of EZN, is the fact that the SEEG signal may not vary monotonously with respect to the size of the activated cortical patch while a monotonic variation seems to be more plausible for simulated scalp-EEG signals (Cosandier-Rimele et al., 2007b). Such a very high sensitivity of SEEG to the surrounding geometry of the brain represents a counterpart to its very high spatial specificity.

### 4.3 Effects of local propagation

While the absence of local coupling modeling during the NMM inversion did not really impact the inference performance in general, we showed one specific example where it had a clear and undesired effect, namely an overestimation of the excitability. Very importantly, this may translate in the context of presurgical planning into the misidentification of a candidate region for resection. This indicates that local coupling must be considered very carefully during the inversion procedure. And indeed, preliminary results of such approaches already showed promising results (Fig. 7G).

### 4.4 Limitations

#### 4.4.1 Simulated data

The comparison between conditions lc_none and lc_0 was introduced to study the specific effect of the forward gain matrix while conditions lc_2 and lc_4 carried the effects of both the gain matrix and the local coupling. To focus on the specific effects of the local coupling, one option was to modify the NMM used for the inversion by artificially assigning the activity of each region node to the set of vertex nodes associated to the region, and thus enabling the use of an identical gain matrix for both simulation and inversion. We did not follow this option because results showed that the reduction of performance was essentially imputable to the gain matrix and because the main purpose of the study was the evaluation of the NMM per se, not its refinement.

#### 4.4.2 Cortical and subcortical regions

In all spatial configurations, we build EZN and PZN only from neocortical regions and did not select subcortical regions, which were systematically considered as point-like structures (NMM) in both the simulation and the inversion procedures. The reason for this is that there is no consensus in the community on how to compute the forward solution for subcortical structures. Given that the effects we observed for neocortical regions should be comparable, but also possibly even more pronounced, for subcortical structures, it remains very important to address such question in the future, and more so as subcortical structures are very often involved in EZN. Our team is currently working in this direction for the particular case of the hippocampus which, despite its complex organization, allows a surfacic representation approach similar to that of neocortical structures. This should enable its short-term inclusion as a spatially extended brain structure and result in a more accurate NFM approach.

#### 4.4.3 Forward model

In this study, we only considered the simple infinite homogeneous model (IHM) for the forward model of NFM because previous studies (Caune et al., 2014;Cosandier-Rimele et al., 2006,2007a) have shown that when SEEG contacts were “close to the epileptic foci and relatively far from the skull” (Caune et al., 2014), the IHM and the one-sphere model provided solutions relatively similar to the ones of a finite element model (FEM). That being said, the use of more realistic and advanced SEEG forward models, for instance based on anisotropic FEM (Bangera et al., 2010;Medani et al., 2023), needs to be addressed in the future, and especially in the context of presurgical planning (Section 4.5). At the same time, we also should not forget that not only the type of forward model (boundary element/FEM, isotropic/anisotropic) plays an important role but the precise localization of the SEEG electrodes and contacts in anatomical images is also essential (Zwick et al., 2022).

#### 4.4.4 Inference techniques

In this study, no particularly informative prior was set for regions of the EZN and PZN and we used a simple inference technique, that is, the quasi-Newton optimization algorithm implemented in Stan’s probabilistic programming language. Because the algorithm is pretty sensitive to its random seed (see the multimodal distributions of GOF onFigs. 3and4), we removed the less effective optimizations, probably due to local minima, with a threshold on the GOF. This resulted in estimates of EZN/PZN with high confidence, while estimates of NIZ were pretty similar to their weakly informative prior (Figs. 3and4). This in turn justified the computation of classification metrics based on point estimates (median of the posterior distributions) rather than full posterior distributions.

It is true that more advanced techniques would definitely have provided better estimation performance, and in particular for the configuration EZN = {multiple regions}. Indeed, well-defined prior combined with Markov Chain Monte Carlo (MCMC) sampling have shown to be determinant when fitting both simulated or empirical data (Hashemi et al., 2020,^2021^,^2023^;Jha et al., 2022;Vattikonda et al., 2021;Wang et al., 2023). In particular, for such fully Bayesian techniques (MCMC, or Simulation Based Inference) which estimate the full posterior distributions, there is no additional need to consider the GOF which is taken into account through the likelihood term (Bayes theorem,Section 2.5.2). With that said, Posterior Predictive Check is still one common diagnostic of Bayesian techniques, similar to the GOF, which is important to perform to check if the fitted model is compatible with the observed data.

However, the purpose here was not to look for the best possible estimation results, but rather to have a simple framework in order to compare between the different conditions in terms of spatial resolution and to really highlight the limitations due to the usage of NMM. Also, given the total number of estimations, the computation time related to MCMC sampling could have been a limiting factor.

## 5 Conclusions and Future Directions

It is fundamental to evaluate the tools and their limitations for good research in epilepsy (Litt, 2022) and especially when they are dedicated to a clinical usage. The estimation of EZN using VEP based on NMM already gives very acceptable results. Still, even in simplest case, the F1 score is not perfect (Fig. 5A) and according to local brain geometry, some regions may be misclassified. We therefore make the hypothesis that an estimation procedure fully based on NFM defined with a good spatial resolution (close to the mm^2^and independent of any parcellation), embedding a realistic forward model (Section 4.4) and implementing local propagation, may be required to obtain more accurate estimations, at least in some specific configurations. Such a tool will be available in the near future (Vattikonda et al., 2023). It will enable the straightforward comparison between NMM and NFM inversions based on a same shared (simulated and empirical) dataset and provide the ideal and rigorous framework to draw new conclusions about the relative benefits of NMM and NFM in estimating the EZN. In addition, flexible NFM inversion itself will of course offer the opportunity to compare different forward models as well as bring new light on the role and importance of local propagation. In the context of clinical translational research, it will also be essential in a future study to evaluate how such an NFM approach could extend the conclusions of previous retrospective studies (Makhalova et al., 2022;Wang et al., 2023), and especially, in which circumstances it could help to predict and achieve better surgical outcomes. Clearly, this step forward will find immediate applications, such as the development of complementary non-invasive diagnostic tools, for instance, based on electroencephalography or magnetoencephalography for which an accurate forward problem is also a required component, and the exploration of innovative therapeutic solutions, such as, for instance, non-invasive stimulation.

## Supplementary Material

Supplementary Material

## Data Availability

Simulated data and code supporting the findings of this study are available from the authors on reasonable request.
