# Supplementary material for: Effects of the spatial resolution of the Virtual Epileptic Patient on the identification of epileptogenic networks
Source: Imaging Neurosci (Camb). 2024 May 8;2:imag-2-00153. doi: 10.1162/imag_a_00153 (PMC12247570; doi:10.1162/imag_a_00153)
Supplement: Supplementary Material [file imag_a_00153-supp.pdf]

Effects of the spatial resolution of the Virtual Epileptic Patient on the identification of epileptogenic networks

Supplementary Material

Supplementary Material contain four sections :

1 Patients and clinical hypothesis 1
2 Goodness of fit 2
3 Estimated parameters 4

1 Patients and clinical hypothesis

	EZ hypothesis
id001	Right-Occipito-temporal-sulcus^{PZ}, Right-Rhinal-cortex^{EZ}, Right-STS-anterior
id002	Left-Fusiform-gyrus^{PZ}, Left-Occipito-temporal-sulcus^{EZ}, Left-Rhinal-cortex, Left-T2-posterior, Right-Collateral-sulcus, Right-Rhinal-cortex
id003	Right-Anterior-cingulate-cortex, Right-F1-mesial-prefrontal, Right-Gyrus-rectus, Right-ITS-anterior, Right-T2-anterior^{EZ}, Right-T2-posterior^{PZ}
id004	Right-Temporal-pole
id007	Right-Rhinal-cortex
id008	Left-Collateral-sulcus^{EZ}, Left-Rhinal-cortex^{PZ}
id010	Left-Rhinal-cortex
id013	Left-Anterior-cingulate-cortex^{PZ}, Left-F1-lateral-prefrontal, Left-Gyrus-rectus^{EZ}, Left-Orbito-frontal-cortex^{PZ}

id017	Left-Collateral-sulcus, Left-Parahippocampal-cortex, Left-Rhinal-cortex^{EZ}, Left-STS-anterior, Left-T2-anterior, Left-Temporal-pole^{PZ}
id018	Left-Angular-gyrus, Left-Intraparietal-sulcus^{EZ}, Left-Superior-parietal-lobule-P1^{PZ}, Left-Supramarginal-posterior, Left-T1-lateral-posterior, Left-T1-planum-temporale
id022	Left-Intraparietal-sulcus^{PZ}, Left-Postcentral-gyrus, Left-Postcentral-sulcus, Left-Superior-parietal-lobule-P1^{EZ}
id038	Left-Occipito-temporal-sulcus, Left-Rhinal-cortex, Left-T2-anterior, Left-T3-anterior, Right-Occipito-temporal-sulcus, Right-Rhinal-cortex^{EZ}, Right-T3-anterior^{PZ}, Right-Temporal-pole^{PZ}
id039	Right-Collateral-sulcus^{PZ}, Right-F3-pars-opercularis, Right-Orbito-frontal-cortex, Right-Rhinal-cortex^{EZ}, Right-T2-anterior, Right-Temporal-pole^{PZ}
id040	Left-Collateral-sulcus, Left-Orbito-frontal-cortex, Left-Parahippocampal-cortex, Left-Rhinal-cortex, Left-STS-anterior^{EZ}, Left-STS-posterior^{PZ}, Left-Supramarginal-posterior, Left-Temporal-pole

Table 1 List of the 14 epileptic patients included in the study along with the individual clinical hypotheses. Clinical hypotheses contain the list of regions suspected to be part of EZN or PZN. They were provided by the clinicians of La Timone Hospital in Marseille based on the presurgical evaluation of each patient, including the computation of an epileptogenicity index for each brain region (Bartolomei et al., 2008). Regions refer to the parcels of the VEP anatomical atlas (Wang et al., 2021). Regions identified with an upper index $^{EZ/PZ}$ are the ones chosen for the specific spatial configuration type EZN = {1 region}, PZN = {1 or 2 regions}.

2 Goodness of fit

Goodness of fit (GOF) measures how well the SEEG signals predicted by the statistical model fit the SEEG signals observed :

$$GOF = \frac{explained\_variance}{total\_variance} = \frac{total\_variance - unexplained\_variance}{total\_variance}$$
$$= 1 - \frac{unexplained\_variance}{total\_variance} = 1 - \frac{variance(seeg\_obs - seeg\_pred)}{variance(seeg\_obs)}$$

where $seeg\_obs$ and $seeg\_pred$ are two-dimensional SEEG signals (with dimensions time and SEEG contacts). The variance of SEEG signals is computed as the sum across all contacts :

$$variance(seeg) = \sum_k variance(seeg_k)$$

where $seeg_k$ is the SEEG signal of contact k.

GOF is always smaller than 1 and 1 indicates a perfect fit.

3 Estimated parameters

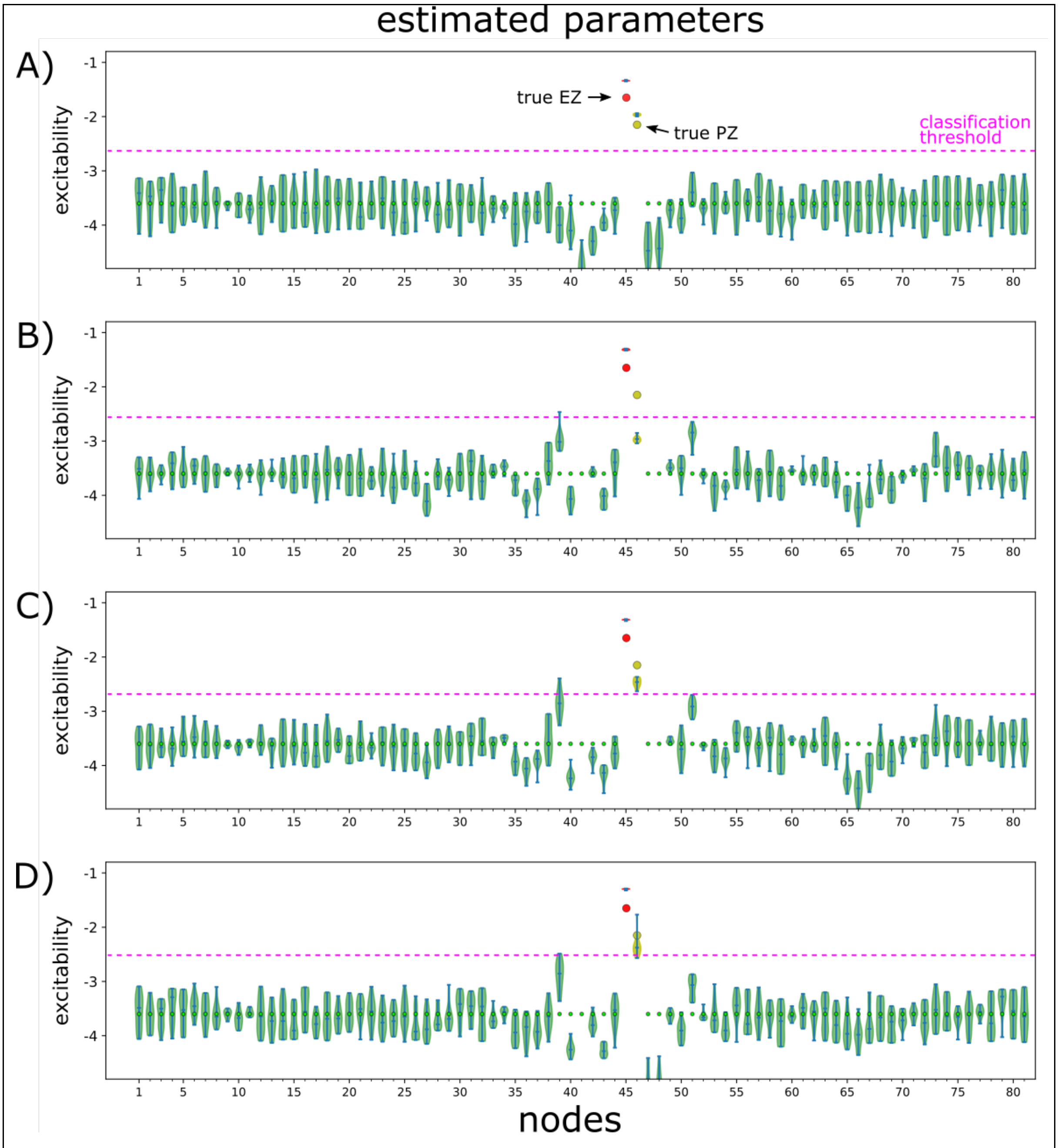

Supplementary Fig. 1 Estimated parameters for patient id003 and EZN = {Right-T2-Anterior}, PZN = {Right-T2-posterior}. This figure refers to Fig. 3 in the main text.

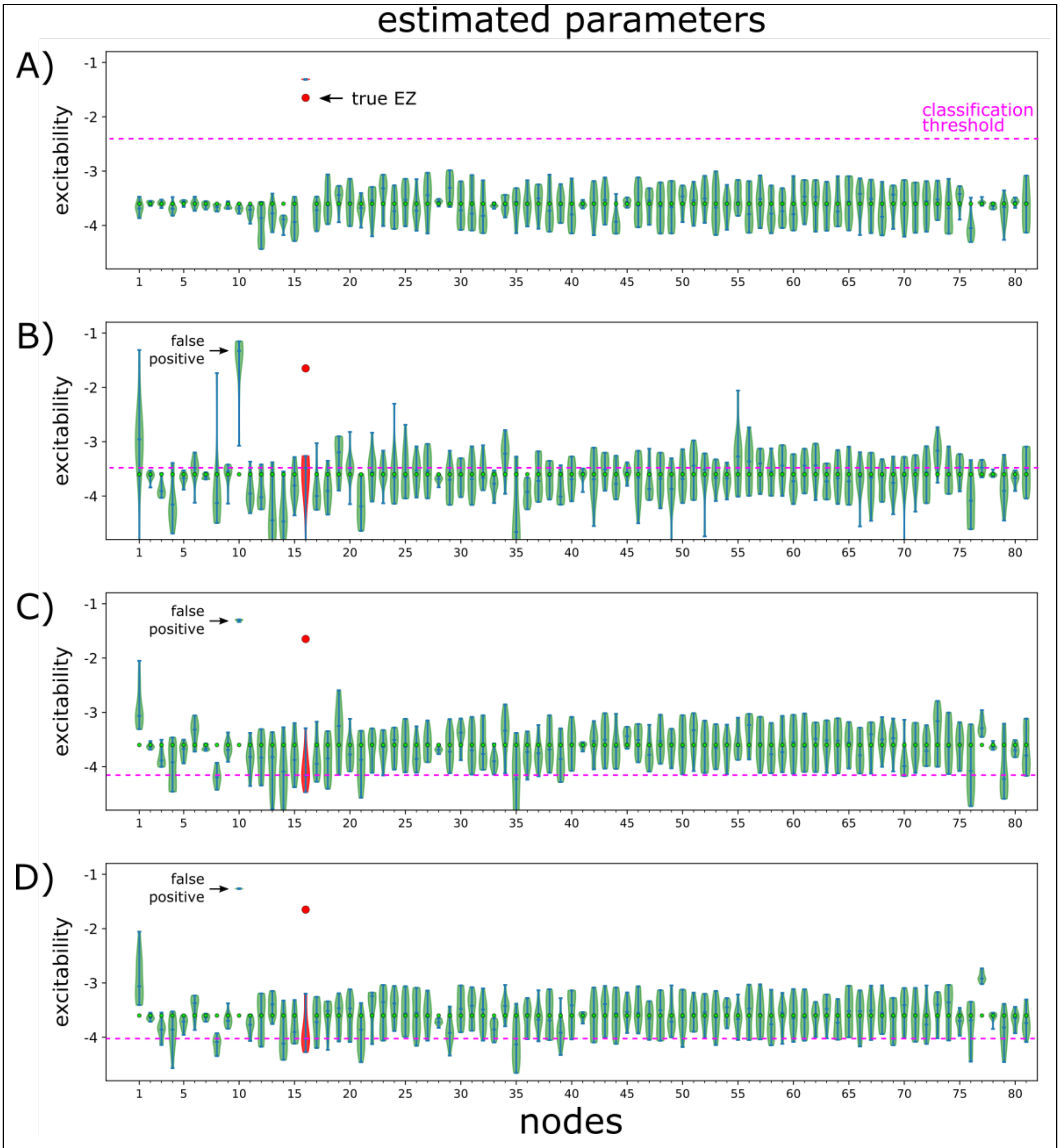

Supplementary Fig. 2 Estimated parameters for patient id013 and EZN = {Left-F1-lateral-prefrontal}. This figure refers to Fig. 4 in the main text.

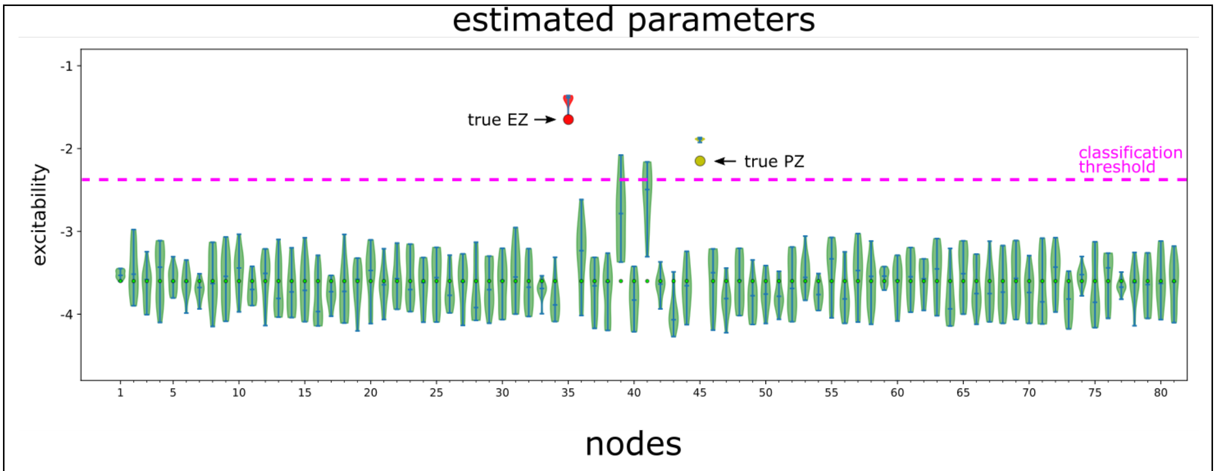

Supplementary Fig. 3 Estimated parameters for patient id017 and EZN = {Left-Temporal-pole}, PZN = {Left-T2-anterior}. This figure refers to Fig. 7 in the main text.
